# Severity-dependent metabolic rewiring in COVID-19 based on untargeted metabolomic profiling of patient plasma

**DOI:** 10.1371/journal.pone.0352437

**Published:** 2026-06-25

**Authors:** Marta Majewska, Mateusz A. Maździarz, Ewa Lepiarczyk, Aleksandra Lipka, Marta Wiszpolska, Beata Moczulska, Elżbieta Łopieńska-Biernat, Piotr Iwanowicz, Piotr Kocbach, Hilde Galtung, Leszek Gromadziński

**Affiliations:** 1 Department of Human Physiology and Pathophysiology, School of Medicine, Collegium Medicum, University of Warmia and Mazury in Olsztyn, Olsztyn, Poland; 2 Department of Botany and Evolutionary Ecology, Faculty of Biology and Biotechnology, University of Warmia and Mazury in Olsztyn, Olsztyn, Poland; 3 Faculty of Dentistry, Institute of Oral Biology, University of Oslo, Oslo, Norway; 4 Department of Cardiology and Internal Medicine, School of Medicine, Collegium Medicum, University of Warmia and Mazury in Olsztyn, Olsztyn, Poland; 5 Department of Biochemistry, Faculty of Biology and Biotechnology, University of Warmia and Mazury in Olsztyn, Olsztyn, Poland; 6 Diagnostyka Medical Laboratories, Olsztyn, Poland; 7 Department of Family Medicine and Infectious Diseases, School of Medicine, Collegium Medicum, University of Warmia and Mazury in Olsztyn, Olsztyn, Poland; University of California Riverside, UNITED STATES OF AMERICA

## Abstract

Coronavirus disease 2019 (COVID-19), caused by the severe acute respiratory syndrome coronavirus 2 (SARS-CoV-2), remains a major global health challenge, characterised by a heterogeneous clinical spectrum. While metabolomic studies have identified disruptions in amino acid, lipid, nucleotide, and energy metabolism during COVID-19, these investigations often lack fine-grained clinical stratification. In this study, we performed untargeted metabolomic profiling of plasma from 25 participants, including five healthy controls and twenty COVID-19 patients classified into four severity groups (COV1–COV4) based on pulmonary involvement and the need for respiratory support. Using ultra-performance liquid chromatography coupled with mass spectrometry (UPLC-MS), 541 metabolites were detected and analysed across all samples. Principal component analysis revealed a progressive metabolic divergence corresponding to disease severity. Monocarboxylic acid dysregulation was predominant in early to moderate cases (COV1–COV3), whereas severe disease (COV4) demonstrated a shift toward pyrimidine metabolism enrichment, consistent with heightened nucleotide turnover driven by viral replication and immune cell proliferation. Phenylalanine metabolism emerged as a consistently enriched pathway in COV1–COV3, suggesting aromatic amino acid perturbations as early markers of metabolic stress and immune activation. In contrast, pyrimidine pathway activation in COV4 could reflect profound systemic metabolic reprogramming associated with critical illness. These findings provide novel insights into COVID-19 pathophysiology, highlighting stage-specific metabolic signatures and potential biomarkers for disease monitoring. Our results support the concept of metabolomics-guided precision medicine, offering a rationale for targeted therapeutic interventions based on disease stage and metabolic phenotype.

## Introduction

Coronavirus disease 2019 (COVID-19), caused by the severe acute respiratory syndrome coronavirus 2 (SARS-CoV-2), remains one of the most significant global health challenges of the modern era. Since its emergence in late 2019, the pandemic has exerted unprecedented pressure on healthcare systems worldwide. As of mid-2025, more than 700 million confirmed cases and over 6 million deaths have been reported globally, underscoring its devastating toll. Importantly, beyond the acute phase, approximately one in four survivors experiences post-acute sequelae, commonly termed long COVID, manifesting as prolonged multi-system morbidity. Despite advances in vaccination and treatment, there remains a critical unmet need for biomarkers that can stratify disease risk, predict outcomes, and guide therapeutic strategies in COVID-19.

SARS-CoV-2 infection is known to profoundly rewire host metabolism [1]. Viral replication is supported by hijacking host metabolic pathways, including glycolysis, nucleotide biosynthesis, lipid turnover, and amino acid metabolism. Reviews have highlighted how SARS-CoV-2 manipulates glucose metabolism, mitochondrial activity, and lipid remodeling during cell entry and replication, while simultaneously reshaping immunometabolic regulation and redox balance [2–4]. Metabolomic studies consistently report perturbations across amino acid, lipid, nucleotide, and energy metabolism, with particular emphasis on pathways involving phenylalanine, tryptophan, and pyrimidines [5]. Such alterations appear to reflect both viral demands and host immune responses. Yet, most of the existing investigations have relied on broad severity classifications, typically contrasting mild versus severe disease, thereby overlooking more subtle gradations along the clinical trajectory. Moreover, while dysregulation of pyrimidine metabolism has been implicated in severe COVID-19 due to viral exploitation of *de novo* nucleotide synthesis, this process remains insufficiently characterized at the metabolite level. Several landmark metabolomic studies have shed light on the biochemical reprogramming associated with COVID-19. Shen et al. reported distinct metabolic and proteomic signatures between moderate and severe cases [2]. Su et al. demonstrated a pronounced metabolic inflection point between mild and moderate disease, closely linked to inflammation and immune activation [3]. Furthermore, Roberts et al., employing untargeted LC-MS on 120 serum samples, identified metabolites such as deoxycytidine, ureidopropionate, kynurenine, and acylcarnitines as potential metabolic predictors of disease severity and clinical outcome [6]. On a similar note, Wu et al. reported coordinated alterations in plasma metabolome and lipidome that mirrored disease trajectories [7].

Subsequent investigations have refined these insights by highlighting stage-specific metabolic signatures. For example, Thomas et al. demonstrated that kynurenine and fatty acid metabolism are altered in relation to interleukin-6 (IL-6) levels and renal function [8]. Overmyer et al. conducted large-scale multi-omics profiling in hospitalised patients and found biomolecular signatures strongly associated with COVID-19 severity, including neutrophil activation, dysregulated complement and coagulation cascades, and profound lipid remodelling. Notably, depletion of plasmalogens, HDL components, and plasma gelsolin, together with increased acute-phase proteins, correlated with poor outcomes [9]. Furthermore, Fraser et al. profiled critically ill patients and identified diagnostic and prognostic biomarkers associated with fatal outcomes and identified kynurenine elevation with concomitant arginine depletion as robust diagnostic markers, while the creatinine/arginine ratio emerged as a powerful prognostic indicator of mortality in COVID-19 [10]. Finally, a scoping review by Pimentel et al. synthesized findings from 42 untargeted metabolomic studies, highlighting recurrent perturbations in phenylalanine, tryptophan, glutamine, urea cycle intermediates, redox balance, and mitochondrial function across severity levels [11].

Despite these advances, an important limitation persists: most studies have applied binary stratifications, comparing extremes of disease severity, rather than examining the full clinical spectrum. Such approaches risk obscuring dynamic metabolic transitions that occur progressively from early to critical disease. Consequently, our understanding of metabolic rewiring across incremental stages of COVID-19 remains incomplete, and the opportunity to identify stage-specific biomarkers has been underutilized.

To address this gap, we conducted a finely stratified metabolomic analysis of plasma from COVID-19 patients, grouped into four clinically defined severity categories (COV1–COV4) based on radiological findings and clinical outcomes, alongside healthy controls. Using untargeted ultra-performance liquid chromatography coupled with mass spectrometry (UPLC-MS). This approach enabled us to capture a progressive metabolic trajectory, revealed by principal component analysis, and to uncover stage-dependent enrichment of distinct pathways.

Our study not only corroborates but also extends prior observations by delineating stage-specific metabolic phenotypes across the full clinical spectrum of COVID-19. These findings provide novel insights into the pathophysiology of SARS-CoV-2 infection, suggest potential biomarkers for risk stratification, and highlight metabolic pathways that could serve as therapeutic targets. Ultimately, this work contributes to the growing evidence base for metabolomics-guided precision medicine, illustrating how comprehensive biochemical profiling can inform stage-tailored interventions in infectious diseases.

## Materials and methods

### Patients

#### Sample acquisition and patient characterization.

Peripheral blood samples were collected from a cohort of 25 patients, consisting of five healthy control donors (CTR; age range: 39–43 years) and twenty patients with confirmed coronavirus disease 2019 (COVID-19; COV). Patient recruitment took place between 25 January 2021 and 15 May 2021 (Resolution No. 3/2021), during a period of increased SARS-CoV-2 transmission in Poland, at the Clinical Department of Communicable Diseases in Ostróda and the Department of Cardiology and Internal Medicine (COVID-19 Unit) at the University Clinical Hospital of the University of Warmia and Mazury in Olsztyn. All participants provided written informed consent, confirmed by their signature, prior to study enrollment. The study cohort comprised 13 male and 7 female participants, reflecting the sex distribution typically observed among hospitalized COVID-19 patients. Patients ranged in age from 41 to 79 years. Patients were stratified into four subgroups (COV1–COV4; n = 5 in each group) according to the extent of pulmonary involvement on computed tomography (CT) and the need for respiratory support. This stratification was adapted from the Radiographic Assessment of Lung Oedema (RALE) score described by Wong et al. [12]. In the RALE system, each lung is independently scored from 0 to 4 based on the percentage of involvement by ground-glass opacities or consolidations (0 = none, 1 = < 25%, 2 = 25–50%, 3 = 50–75%, 4 = > 75%), yielding a total score of 0–8. Based on this framework, subgroup COV1 was defined as <25% involvement (RALE 0–1), COV2 as 25–50% (RALE 2), and COV3 as 51–100% (RALE 3–4). Subgroup COV4 comprised patients with the most severe disease requiring critical respiratory support, all of whom experienced fatal outcomes within 11–50 days of hospital admission. Pulmonary involvement was quantified by CT as the estimated proportion of lung parenchyma affected by ground-glass opacities (GGOs; hazy areas with preserved vascular markings reflecting partial alveolar filling), crazy-paving patterns (ground-glass opacities with superimposed septal thickening giving a tiled appearance, indicating combined alveolar and interstitial involvement), or consolidations (dense opacities obscuring vessels due to complete alveolar filling). Molecular testing (RT-qPCR) confirming SARS-CoV-2 infection was performed upon hospital admission, and blood samples were collected immediately thereafter, within the first 2 hours of hospitalisation. All samples were obtained prior to the initiation of corticosteroid therapy or any major clinical interventions, ensuring that metabolomic profiles reflected the early phase of hospital-managed disease and minimizing the influence of therapeutic factors. All COVID-19 patients were treated according to the institutional clinical management protocol in place during the study period (January–May 2021). Notably, this timeframe represented a high-transmission phase of SARS-CoV-2 in Poland, with the B.1.1.7 (Alpha) documented as the prevailing circulating variant. Standard therapy included supportive care, anticoagulant treatment, corticosteroids when clinically indicated, and supplemental oxygen depending on disease severity. The study was performed in accordance with the Declaration of Helsinki and was approved by the Bioethics Committee of the Warmia-Mazury Medical Chamber (OIL.3/2021/Bioet) in Olsztyn, Poland. All participants gave written informed consent (confirmed by their signature) to participate in the study.

#### Diagnostic criteria and exclusion parameters for COVID-19 patients.

SARS-CoV-2 infection was diagnosed through reverse transcription-polymerase chain reaction (RT-qPCR) analysis of nasopharyngeal swab specimens, confirming the presence of viral genes. RT-qPCR assays were conducted using the COVID-19 Real-Time Multiplex RT-PCR Kit (Labsystems Diagnostics OY, Vantaa, Finland), which enables simultaneous detection of the ORF1ab, N, and E genes of the SARS-CoV-2 genome. All RT-qPCR procedures adhered to the manufacturer’s protocol. Quantitative analysis of amplification curves was performed using a QuantStudio™ 5 Real-Time PCR System.

Inclusion criteria for COVID-19 patients necessitated a confirmed positive SARS-CoV-2 PCR test and a clinical diagnosis of COVID-19 requiring hospital admission. Exclusion criteria included neoplasia, autoimmune disorders, immunosuppression, immunodeficiency, or human immunodeficiency virus (HIV) infection.

#### Characterization and inclusion criteria for healthy controls.

The control group consisted of healthy volunteers who tested negative for SARS-CoV-2 and exhibited no clinical indications of respiratory tract infections or pulmonary pathologies. Inclusion criteria for the control group were confirmed via a screening questionnaire and included no history of travel to high-risk areas, no SARS-CoV-2 vaccination, no documented or suspected SARS-CoV-2 exposure within the preceding 14 days, absence of active upper or lower respiratory tract infection or any other acute illness at the time of blood collection, and no history of severe chronic diseases, including immune disorders. All healthy volunteers additionally underwent clinical examination and laboratory screening to exclude infection or inflammation prior to inclusion. None of the participants reported fever, respiratory symptoms, or recent vaccination within the previous 14 days. Basic biochemical and hematological parameters, including C-reactive protein (CRP), leukocyte counts, and liver enzyme activities (ALT, AST), were measured and found to be within normal reference ranges for all individuals. These findings confirmed the absence of systemic inflammation or acute disease at the time of sampling. Control blood samples were collected under standardized conditions from fasting participants in the morning hours. All control donors provided written informed consent prior to participation and confirmed that they were not using any prescription medications or dietary supplements that could affect metabolic or inflammatory parameters.

#### Blood sample processing for untargeted metabolomics.

Three millilitres (3 mL) of whole blood were collected from each participant into K₂-EDTA vacutainer tubes (Greiner Bio-One GmbH, Austria). Tubes were gently inverted 8–10 times immediately after collection and placed on wet ice until processing. Within 30 minutes of venipuncture, samples were centrifuged at 1,500 g for 10 minutes at 4 °C. The plasma fraction was carefully aspirated, aliquoted into low-binding polypropylene tubes (100–200 µL), snap-frozen in liquid nitrogen, and stored at −80 °C until metabolomics analysis.

#### Biochemical and hematological assessment of blood sample parameters in COV1-COV4 patients.

Blood samples from the COV1, COV2, COV3, and COV4 cohorts were subjected to the following analyses: C-reactive protein (CRP) levels (mg/L), D-dimer concentrations (ng/mL), leukocyte counts (k/μL), alanine aminotransferase (ALT) activity (U/L), and aspartate aminotransferase (AST) activity (U/L). Comparative analyses between the cohorts were performed using the Kruskal–Wallis one-way analysis of variance by ranks with the R stats library. Where statistically significant differences were identified, Dunn’s post hoc test was applied for pairwise group comparisons between independent cohorts (e.g., COV1 vs COV2, COV2 vs COV3, COV3 vs COV4). To account for multiple testing, p-values were adjusted using the Benjamini–Hochberg (BH) procedure to control the false discovery rate. An adjusted p-value (padj) of less than 0.05 was considered statistically significant.

#### Chest computed tomography.

Chest CT served as the primary diagnostic tool for patient management during the initial phase of the severe acute respiratory syndrome coronavirus 2 (SARS-CoV-2) pandemic. Institutional protocol dictated hospital admission criteria, which included a peripheral arterial oxygen saturation level of ≤93%. This threshold is consistent with NIH definitions of severe COVID-19 (SpO₂ < 94% on room air) and falls within the range of oxygen saturation values used in clinical practice [13]. WHO guidelines apply a more stringent threshold (SpO₂ < 90%); however, intermediate values (90–94%) are commonly used for clinical stratification in hospitalized patients [14]. All patients underwent a non-contrast chest CT scan in the supine position using a Toshiba Medical System, Aquilion Prime type TSX-303A/BK. Scan parameters included a tube voltage of 120–135 kV, tube current of 530–600 mA, 160 layers, and 80 rows. Image analysis was performed using Osirix MD 11.0™ software (Pixmeo Company, Bernex, Switzerland) by two independent radiologists experienced in chest CT. These radiologists were blinded to the reverse transcription-polymerase chain reaction (RT-PCR) results of individual patients. Qualitative assessment of the chest CT scans focused on identifying opacity types, characterizing their morphology and distribution, and quantifying the percentage of involved lung parenchyma.

### Untargeted metabolomics

#### Sample preparation.

Samples were thawed and a 100 μL aliquot of each sample was transferred into a designated sample tube. Subsequently, 300 μL of methanol was added, and the mixture was vortexed for 30 seconds. All samples were incubated at −20°C for 1 hour, followed by centrifugation at 12,000 rpm and 4°C for 15 minutes. Finally, 200 μL of the resulting supernatant was combined with 5 μL of DL-o-Chlorophenylalanine (0.5 mg/mL, Merck) and transferred to a vial for liquid chromatography-mass spectrometry (LC-MS) analysis. To assess the reliability of the analytical workflow, quality control (QC) samples were prepared by pooling equal aliquots of all study samples. QC samples were subjected to identical handling and extraction procedures, including a single freeze–thaw cycle, as applied to analytical samples. All plasma samples (including QC) were thawed, extracted, and prepared within a single analytical session to minimize batch-related variability. QC samples were injected regularly throughout the LC–MS sequence to monitor signal stability, retention time (RT) reproducibility, and instrument performance.

QC samples were analyzed in both positive and negative ionization modes at regular intervals throughout the sequence. The ion features of the QC injections were used to calculate the relative standard deviation (RSD) distribution, which provides a quantitative measure of analytical stability. As shown in S1 Fig in S1 File (derived from the analytical report), the majority of detected ion features exhibited %RSD values below 30%, confirming excellent reproducibility of the LC–MS system. The stability and reproducibility of the analytical process were further confirmed by principal component analysis (PCA) of QC samples, which demonstrated consistent clustering and low variance (S1 Fig in S1 File). These findings validate the robustness of the analytical workflow and support the reliability of the metabolomic comparisons presented in Fig 3C. A total of four distinct comparative analyses were performed. The first analysis involved a comparison of the Control group (CTR) samples with the COV1 samples. The second analysis compared CTR samples with COV2 samples. The third analysis contrasted CTR samples with COV3 samples. Finally, the fourth analysis compared CTR samples with COV4 samples.

#### Ultra-performance liquid chromatography–mass spectrometric (UPLC-MS) conditions.

Chromatographic separation was performed using an UltiMate 3000 LC system coupled to a Q Exactive mass spectrometer (Thermo Fisher Scientific, Bremen, Germany), equipped with an electrospray ionization (ESI) source operating in both positive and negative ionization modes. The LC system was fitted with an ACQUITY UPLC HSS T3 column (100 × 2.1 mm, 1.8 μm particle size; Waters). The mobile phase consisted of solvent A (0.05% formic acid in water) and solvent B (acetonitrile), using the following gradient: 0–1 min, 95% A; 1–12 min, 95–5% A; 12–13.5 min, 5% A; 13.5–13.6 min, 5–95% A; 13.6–16 min, 95% A. The flow rate was 0.3 mL·min ⁻ ¹, column temperature 40°C, and autosampler temperature 4°C. The Q Exactive mass spectrometer was operated in full-scan (MS¹) mode for untargeted profiling, combined with combined with MS/MS fragmentation for metabolite annotation. The mass scan range was m/z 70–1050, and the resolving power was set to 70,000 FWHM (at m/z 200). The automatic gain control (AGC) target was 3 × 10⁶ ions, and the maximum injection time was 100 ms. The ESI source parameters were as follows. For ESI(+): spray voltage 3.0 kV, heater temperature 300°C, sheath gas flow 45 arb, auxiliary gas 15 arb), sweep gas 1 arb, capillary temperature 350°C, and S-lens RF level 30%. For ESI(–): spray voltage −3.2 kV, heater temperature 300°C, sheath gas flow 45 (arb), auxiliary gas 15 arb, sweep gas 1 arb, capillary temperature 350°C, and S-lens RF level 60%. Higher-energy collisional dissociation (HCD) was applied at a normalized collision energy (NCE) of 30% for MS/MS data acquisition. Data were acquired using Thermo Xcalibur 4.1 software and processed in Compound Discoverer 3.0 (Thermo Fisher Scientific). All parameters were verified based on the official analytical report (Creative Proteomics, Report ID: CPMT12062104), ensuring that the reported settings accurately reflect the conditions used for metabolomic data acquisition in this study. Data were acquired in both ESI positive and negative ionization modes, and features from both datasets were merged after RT alignment and peak area normalization. Chromatographic alignment was performed independently for datasets acquired in ESI(+) and ESI(−) ionization modes, based on matching of m/z and RT features across all samples. Due to differences in ionization behavior, feature detection, and independent data processing between modes, feature annotations may not always directly correspond across modes, and no cross-mode alignment was performed. Due to differences in ionization efficiency, the same metabolite may be detected in both modes and treated as separate features during statistical analysis. The combined dataset included features detected in either ionization mode, providing complementary coverage of polar and nonpolar metabolites. This approach ensured a broad representation of biochemical changes associated with COVID-19 severity.

#### Quantification and differential analysis of metabolites.

Raw data were acquired and aligned using Compound Discoverer (version 3.0, Thermo Scientific) based on the m/z values and retention times of the detected ion signals. Ions from both electrospray ionisation ESI- and ESI+ modes were merged and subsequently imported into SIMCA-P software (version 14.1) for multivariate statistical analysis. Subsequently, supervised regression modelling was performed using either Partial Least Squares Discriminant Analysis (PLS-DA) or Orthogonal Partial Least Squares Discriminant Analysis (OPLS-DA) to identify potential biomarkers. Statistical comparisons between each COVID-19 severity group (COV1–COV4) and the healthy controls were performed using a two-tailed Student’s t-test. To correct for multiple hypothesis testing, p-values were adjusted using the Benjamini–Hochberg false discovery rate (FDR) procedure. Adjusted p-values (padj) were used to identify significantly dysregulated metabolites (|log₂FoldChange| ≥ 1.0 and padj < 0.05). Metabolites meeting these thresholds were considered significantly altered and were further validated based on their variable importance in projection (VIP) scores derived from PLS-DA/OPLS-DA models. Metabolites were further categorized into major biochemical classes, including amino acids, lipids, and nucleotides, based on annotations from the Human Metabolome Database (HMDB; www.hmdb.ca) and pathway classification using MBROLE3 integrating Kyoto Encyclopedia of Genes and Genomes (KEGG) information. Significant KEGG and chemical annotations were defined as those with padj < 0.05. Metabolite identification was based on accurate-mass matching and, where available, MS/MS fragmentation data, using database-driven annotation workflows. In accordance with standard untargeted metabolomics practices, the reported metabolites should be considered as putative annotations (Metabolomics Standards Initiative level 2), rather than fully confirmed identifications based on authentic reference standards.

### Visualization

The PCA plot was generated using the factoextra v.1.0.7 library. The Venn diagram was created with the ggvenn v.0.1.10 library, and circular charts were constructed using the circlize v.0.4.16 library. Finally, both volcano plots and bar plots were produced with the ggplot2 v.3.5.2 library.

## Results

### Clinical characteristics of the study cohort and radiological findings

**Fig 1:**
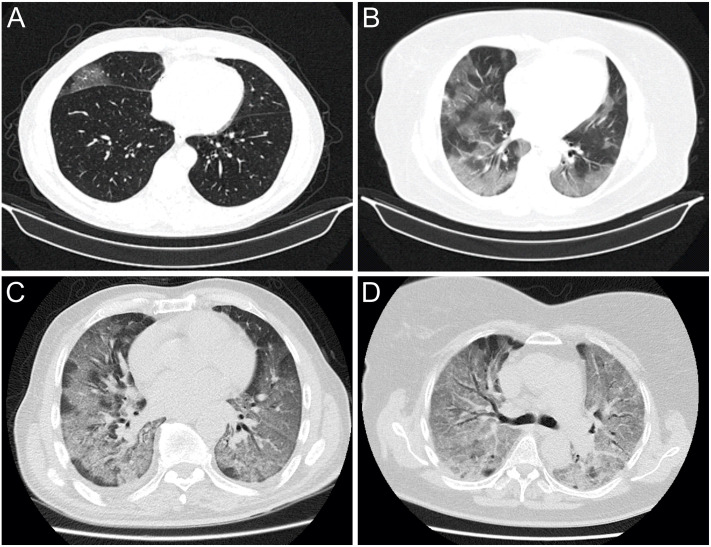
Noncontrast axial high-resolution computed tomography (CT) of the chest images of inflammatory changes characteristic of COVID-19 disease. **A COV1 group.** Peripheral ground-glass opacities limited to small areas (15% of lung parenchyma) in the COV1_3 patient. **B. COV2 group**. Bilateral ground-glass opacities, more pronounced in the right lung (50% of lung parenchyma) in the COV2_4 patient. **C. COV3 group**. Extensive bilateral ground-glass opacities with superimposed crazy-paving pattern and discrete consolidations (85% of lung parenchyma) in the COV3_4 patient. **D. COV4 group.** Diffuse, near-complete opacification of both lungs with extensive ground-glass opacities and consolidations (90% of lung parenchyma) in the COV4_1patient.

Subgroup COV1 (<25% pulmonary involvement) consisted of patients aged 50–70 years (four males, one female), all of whom did not require respiratory support. The median pulmonary involvement in this subgroup was 8% (range: 2–15%). COV2 (25–50% involvement) included patients aged 49–70 years, predominantly female (four females, one male), with a median pulmonary involvement of 35% (range: 30–50%). COV3 (51–100% involvement) comprised patients aged 41–67 years (four males, one female), with a median pulmonary involvement of 77% (range: 60–85%). Subgroup COV4 represented the most severe cases, consisting of patients aged 66–79 years (four males, one female), all requiring critical respiratory support and experiencing fatal outcomes within 11–50 days after hospital admission. Pulmonary involvement in this subgroup had a median of 78% (range: 60–90%) (Table 1, Fig 1). Pairwise age comparisons revealed no statistically significant differences among the COVID-19 severity groups (COV1–COV4) after adjustment for multiple testing (Benjamini–Hochberg procedure, padj > 0.05 for all comparisons). In contrast, patients in the COV4 group were significantly older than healthy controls (CTR vs. COV4, padj < 0.001). No other CTR–COV comparisons remained significant after p-value adjustment.

**Table 1:** Clinical characteristics of study participants.

Group	Patient	% of pulmonary involvement	Sex	Age	Clinical outcome
**CTR**	CTR_1	–	F	38	Healthy control
	CTR_2	–	F	43	
	CTR_3	–	M	39	
	CTR_4	–	M	42	
	CTR_5	–	M	39	
**COV1 (<25% of pulmonary involvement with none requiring respiratory support)**	COV1_1	15%	M	69	Recovered; discharged
	COV1_2	3%	M	70	
	COV1_3	15%	M	64	
	COV1_4	7%	F	66	
	COV1_5	2%	M	50	
**COV2 (25–50% of pulmonary involvement with none requiring respiratory support)**	COV2_1	30%	F	64	
	COV2_2	35%	F	70	
	COV2_3	30%	F	49	
	COV2_4	50%	F	69	
	COV2_5	30%	M	65	
**COV3 (51–100% of pulmonary involvement with none requiring respiratory support)**	COV3_1	70%	M	46	
	COV3_2	60%	F	67	
	COV3_3	85%	M	41	
	COV3_4	85%	M	66	
	COV3_5	85%	M	54	
**COV4 (requiring critical respiratory support)**	COV4_1	90%	F	71	Fatal outcome (11–50 days post-admission)
	COV4_2	70%	M	73	
	COV4_3	70%	M	78	
	COV4_4	85%	M	79	
	COV4_5	60%	M	66	

The table presents detailed demographic and clinical data for the study groups, including healthy controls (CTR) and four COVID-19 patient cohorts (COV1–COV4), categorized according to the extent of pulmonary involvement and the need for respiratory support. The percentage of pulmonary involvement was assessed by chest computed tomography. For each participant, sex (F – female; M – male) and age (years) are reported.

Across subgroups, quantitative CT assessment demonstrated a progressive increase in the extent of parenchymal involvement: median 8% in COV1, 35% in COV2, 77% in COV3, and 78% in COV4. Mild disease (COV1) was characterized by localized peripheral GGOs, moderate-to-severe disease (COV2–COV3) by confluent and bilateral opacities, and critical illness (COV4) by diffuse GGOs with crazy-paving patterns and consolidations. These findings indicate that radiological severity closely paralleled clinical course, supporting the utility of CT imaging in stratifying and monitoring COVID-19 pneumonia.

### Blood test results

**Fig 2:**
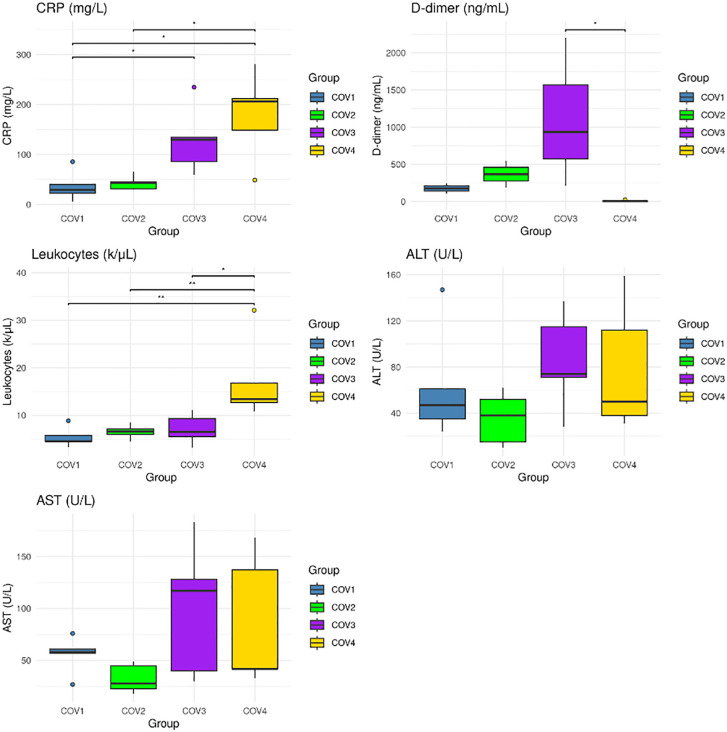
Box plots illustrating the comparison of blood parameters, including CRP, D-dimer, Leukocytes, ALT, and AST. Each plot displays data for four distinct groups of COVID-19 patients, with colors indicating the group: COV1 (blue), COV2 (green), COV3 (purple), and COV4 (yellow). Statistically significant differences are indicated by asterisks (*: 0.01 <= padj < 0.05; **: 0.001 <= padj < 0.01).

Statistically significant differences in C-reactive protein (CRP) [mg/L] levels were observed among patient groups, specifically when comparing COV1 with COV4 (padj = 0.017), COV1 with COV3 (padj = 0.036), and COV2 with COV4 (padj = 0.032). Furthermore, significant discrepancies in leukocyte [k/uL] counts were noted in comparisons between COV1 and COV4 (padj = 0.012), COV2 and COV4 (padj = 0.048), and COV3 and COV4 (padj = 0.043). Additionally, significant differences in D-dimer [ng/mL] concentrations were identified between groups COV3 and COV4 (padj = 0.032). In contrast, indicators such as alanine aminotransferase (ALT) [U/L] and aspartate aminotransferase (AST) [U/L] did not exhibit statistically significant variations. The absence of significant differences in fundamental indicators between groups COV1 and COV2 indicates a high degree of similarity characterizing patients from these two cohorts. Similarly, this situation was observed in the comparison of groups COV2 and COV3, which also suggests their clinical resemblance (S1 Table in S2 File; Fig 2).

### A metabolomic study

Untargeted LC–MS metabolomic profiling was performed in both ESI⁺ and ESI⁻ ionization modes to ensure comprehensive metabolite coverage. Following data alignment, features from both modes were merged into a single integrated dataset, providing complementary detection of polar and nonpolar compounds. The resulting data matrix was used for multivariate and univariate statistical analyses. A complete list of all identified metabolites is provided in Supplementary Tables S2–S5 in S2 File, and their chemical classification is summarised in Supplementary Table S6 in S2 File.

### Differential analysis of metabolites

**Fig 3:**
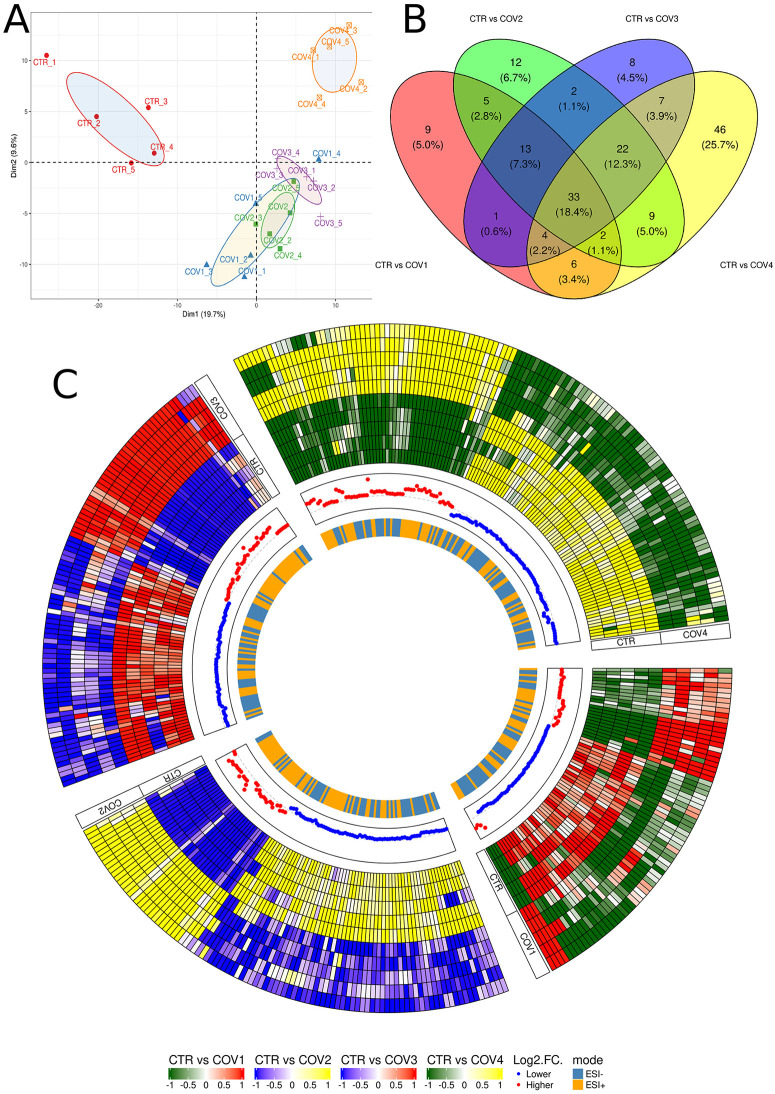
Multidimensional analysis of metabolite profiles in CTR and COV samples. **A.** PCA plot showing the separation of CTR samples (red circles), COV1 (blue triangles), COV2 (green squares), COV3 (purple crosses), and COV4 (orange squares with a cross) in two dimensions: Dim1 (x-axis) and Dim2 (y-axis). **B.** Venn diagram illustrating the number of significant metabolites shared among the comparisons: CTR vs COV1 (red), CTR vs COV2 (green), CTR vs COV3 (blue), and CTR vs COV4 (yellow). **C.** Circos plot comparing significant metabolites. The outermost track represents a heatmap for the following comparisons: CTR vs COV1 (green-red scale), CTR vs COV2 (blue-yellow scale), CTR vs COV3 (blue-red scale), and CTR vs COV4 (green-yellow scale). The innermost track indicates whether metabolite levels are higher in CTR (blue dots) or higher in COV1, COV2, COV3, or COV4 (red dots).

Differential analysis was conducted to identify significantly different metabolites between CTR and COV groups. A total of 541 metabolites were analyzed in each comparison. A clear distinction in the metabolic profile was observed between the CTR group and the COV1, COV2, COV3, and COV4 groups through PCA. Furthermore, the COV4 group was also noted to be distinct from the CTR, COV1, COV2, and COV3 groups. It was observed that the COV1, COV2, and COV3 groups clustered closely together on the PCA plot (Fig 3A), which suggested their metabolic similarity. In the CTR vs. COV1 comparison, 73 metabolites were found to be significantly different (Figs 3B and 3C; S2 Table in S2 File; S2 Fig in S1 File). Similarly, 98, 90, and 129 metabolites were found to be significantly different in the CTR vs COV2, CTR vs COV3, and CTR vs COV4 comparisons, respectively (Figs 3B and 3C; S3-S5 Tables in S2 File; S3-S5 Figs in S1 File). Chemical classification analysis was performed to identify the most significant classes of molecules. Monocarboxylic acids or derivatives were found to be the most significant class of metabolites in the CTR vs COV1, COV2, and COV3 comparisons (S6-S8 Figs in S1 File; S6 Table in S2 File). In contrast, organopnictogen compounds were found to be the most significant class of metabolites in the CTR vs COV4 comparison (S9 Fig in S1 File; S6 Table in S1 File).

### KEGG pathway analysis

**Fig 4:**
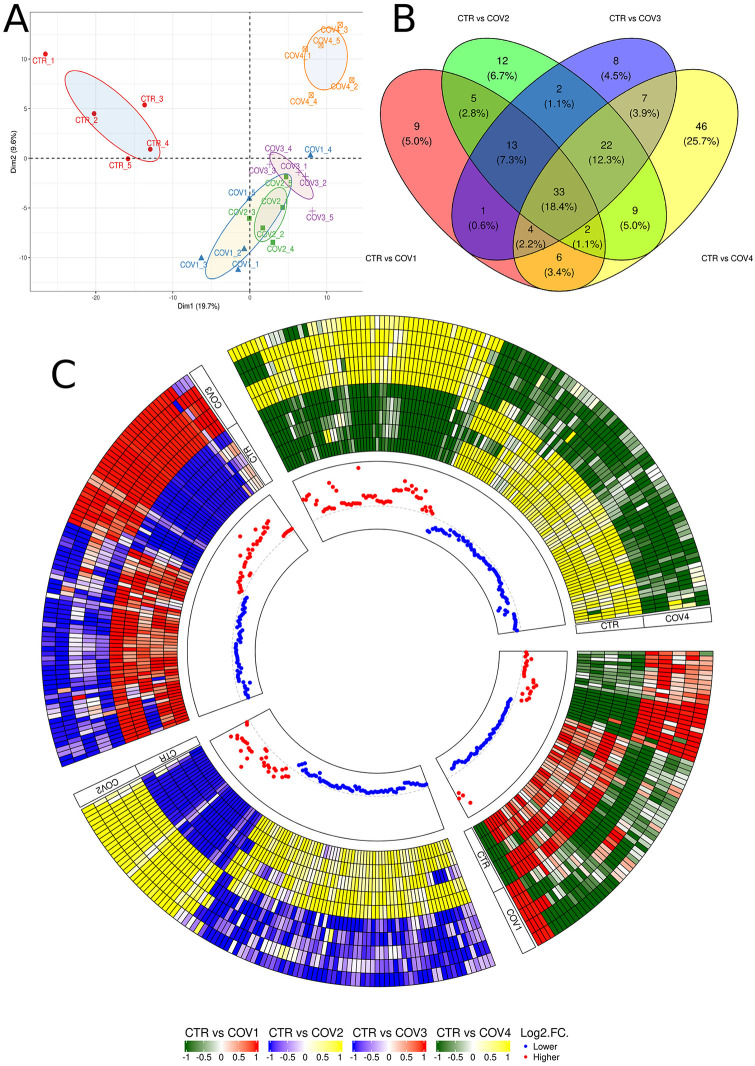
The chord diagram illustrates the statistically significant KEGG pathways identified in several comparisons. Pathways from the CTR vs. COV1 comparison are represented in red, CTR vs. COV2 in green, CTR vs. COV3 in blue, and CTR vs. COV4 in yellow. Each segment of the chart corresponds to a specific KEGG process, identified by a number and its assigned color. For instance, 00240 represents pyrimidine metabolism (purple), 04917 is the prolactin signaling pathway (gray), 01230 refers to the biosynthesis of amino acids (pink), 00360 to phenylalanine metabolism (brown), 00220 to arginine biosynthesis (orange), and 05230 to central carbon metabolism in cancer (slate blue). The connecting lines within the chart indicate the link between a metabolite and its respective pathway.

KEGG pathway enrichment analysis was performed to identify significantly dysregulated pathways across group comparisons. In the CTR vs. COV1 comparison, significant alterations were observed in phenylalanine metabolism (hsa00360), arginine biosynthesis (hsa00220), biosynthesis of amino acids (hsa01230), and central carbon metabolism in cancer (hsa05230) (Fig 4; S7 Table in S2 File).

Similarly, phenylalanine metabolism (00360) and prolactin signaling pathway (04917) were found to be significantly enriched in the CTR vs COV2 comparison (Fig 4; S7 Table in S2 File). In the CTR vs COV3 comparison, phenylalanine metabolism (00360) and biosynthesis of amino acids (01230) were found to be significantly enriched (Fig 4; S6 Table in S2 File). Finally, pyrimidine metabolism (00240) was found to be significantly enriched in the CTR vs COV4 comparison (Fig 4; S7 Table in S2 File).

## Discussion

Our comprehensive profiling of plasma from COVID-19 patients revealed significant metabolomic perturbations associated with disease severity. Principal component analysis (PCA) of 541 metabolites demonstrated a clear stratification between COVID-19 patients and healthy controls, particularly distinguishing severe cases (COV4) from both the control and less severe groups (COV1–COV3) (Fig 3A). The progressive metabolic divergence, especially pronounced in the COV4 cohort, implies a severity-associated metabolic trajectory, likely reflective of increasing systemic dysfunction (Fig 3A). This observation is consistent with results reported by other researchers [15,2]. Differential analysis identified numerous significantly altered metabolites across comparisons, with the highest number in the CTR *vs* COV4 group, indicating an escalating metabolic imbalance with clinical severity (Figs 3B and 3C; S2-S5 Tables in S2 File). Additionally, the present study revealed differences in CRP, leukocyte counts, and D-dimer concentrations, which reflect progressive inflammatory and coagulation disturbances with increasing COVID-19 severity (Fig 1, S1 Table in S2 File). The most pronounced alterations were observed in the COV4 group, indicating a distinct systemic response in critically ill patients. In contrast, the lack of significant changes in ALT and AST suggests limited hepatic involvement in disease progression. Moreover, the similarity between COV1, COV2, and COV3 highlights the clinical and biochemical overlap in early to moderate stages, despite varying pulmonary involvement (Fig 1, S1 Table in S2 File). Age is a well-established risk factor for severe COVID-19 and is associated with baseline metabolic alterations. In the present cohort, however, no statistically significant age differences were observed among the COVID-19 severity groups (COV1–COV4), indicating that the severity-associated metabolic differences identified in this study are unlikely to be driven by age-related bias within the infected population. Although the most severe group (COV4) differed significantly in age compared with healthy controls, this effect was not observed across the remaining COVID-19 groups. Therefore, the observed stepwise metabolic rewiring across disease stages primarily reflects COVID-19 pathophysiology and host response rather than age alone. Nevertheless, the contribution of age-associated metabolic changes to disease severity cannot be excluded and warrants investigation in larger, age-stratified cohorts.

KEGG pathway analysis provided further evidence for the biological significance of the observed metabolic alterations. This analysis consistently demonstrated a clear stratification between COVID-19 patients and healthy controls. Notably, once again it effectively distinguished severe cases (COV4) from both the control group and less severe cases (COV1–COV3). Enrichment of the phenylalanine metabolism pathway (KEGG: 00360) was observed across the COV1–COV3 patient groups. This finding suggests a potential role for aromatic amino acid dysregulation in the pathophysiology of less severe COVID-19 cases. Conversely, the COV4 group was characterized by a significant enrichment in the pyrimidine metabolism KEGG pathway (Fig 4, S7 Table in S2 File).

### Phenylalanine metabolism disturbances in early-stage and moderate COVID-19

Our metabolomic analysis revealed consistent and statistically significant enrichment of the phenylalanine metabolism pathway in COVID-19 patient groups COV1, COV2, and COV3, when compared to healthy controls (Fig 4, S7 Table in S2 File). This repeated enrichment across progressively symptomatic, yet non-critical, cases suggests that alterations in aromatic amino acid metabolism may serve as early metabolic signatures of SARS-CoV-2 infection and contribute to systemic immune dysregulation. Phenylalanine, an essential aromatic amino acid, plays a central role not only in protein synthesis but also as a precursor for tyrosine and downstream catecholamines, including dopamine, norepinephrine, and epinephrine. Its metabolism is primarily regulated by phenylalanine hydroxylase (PAH), which catalyzes its conversion to tyrosine. Dysregulation in this pathway – manifested as elevated circulating phenylalanine or altered levels of its catabolites – has been previously reported in inflammatory and infectious conditions and is increasingly recognized as a marker of metabolic and immune stress [16]. In the context of COVID-19, elevated phenylalanine levels have been consistently associated with disease severity, systemic inflammation, and impaired hepatic function [17].

Importantly, our study revealed that the enrichment of the phenylalanine metabolism pathway in COVID-19 was not driven by changes in phenylalanine itself, but rather by coordinated alterations in multiple aromatic metabolites associated with phenylalanine and tyrosine metabolic networks. These included phenol, cresol, 4-methylcatechol, mandelic acid, hydrocinnamic acid, 2-phenylglycine, salicylic acid, and gentisic acid, which are widely recognized as products of host–microbiome co-metabolism and aromatic amino acid–related biochemical processes [18,19]. Importantly, we emphasize that this enrichment reflects broader perturbations in aromatic amino acid–associated metabolic processes, including host–microbiome co-metabolism and downstream phenolic derivatives, rather than a direct alteration in phenylalanine concentration or flux per se. Thus, the observed pathway enrichment should be interpreted as a disruption of aromatic amino acid–related metabolic networks rather than isolated dysregulation of phenylalanine metabolism alone. In this context, aromatic metabolites derived from phenylalanine and tyrosine metabolism are increasingly recognized as sensitive indicators of immune activation, inflammation, and host–microbiome interactions [20,21].

A progressive pattern was evident across COV1–COV3, consistent with increasing perturbation of aromatic amino acid–associated metabolism during early and moderate disease. In contrast, the absence of phenylalanine pathway enrichment in COV4 coincided with the emergence of pyrimidine metabolism as the dominant enriched pathway, indicating a metabolic shift toward nucleotide turnover and proliferative or catabolic stress in critical illness. This transition suggests that aromatic amino acid–related disturbances may represent an early metabolic response to SARS-CoV-2 infection that becomes masked or superseded by more global metabolic reprogramming at advanced disease stages.

### Pyrimidine metabolism dysregulation in severe covid-19

In contrast to earlier disease stages, KEGG pathway analysis revealed significant enrichment of pyrimidine metabolism (hsa00240) exclusively in patients with severe COVID-19 (COV4) (Fig 4, S7 Table in S2 File), indicating a disease-stage–specific metabolic shift. This enrichment was supported by differential abundance of pyrimidine-related metabolites detected in our dataset, including increased deoxycytidine diphosphate (dCDP) and altered levels of xanthine, alongside depletion of ribose and other central carbon intermediates (Supplemental Table 5).

These changes suggest intensified nucleotide turnover and altered balance between nucleotide synthesis and degradation in severe disease. Importantly, pyrimidine pathway enrichment was not observed in COV1–COV3, indicating that disruption of nucleotide metabolism is a feature of advanced COVID-19 rather than an early response to infection. The emergence of this pathway in COV4 coincided with the loss of phenylalanine metabolism enrichment, supporting a transition from localized aromatic amino acid–associated disturbances to broader metabolic reprogramming involving nucleic acid metabolism.

Pyrimidine nucleotides, comprising uridine, cytidine, and thymidine derivatives, are essential for a variety of biological processes, including RNA and DNA synthesis, glycosylation, phospholipid metabolism, and cellular signaling [22]. SARS‑CoV‑2 infection greatly increases demand for these nucleotides, as the virus hijacks host pyrimidine biosynthesis, disrupting the balance between de novo and salvage pathways. The enrichment of this pathway in the COV4 group suggests intensified host–virus interactions, where elevated synthesis compensates for viral replication and tissue injury [2,23]. Excessive pathway activation may also drive hyperinflammation, as T‑cell proliferation depends on nucleotide synthesis; in severe disease, this becomes maladaptive, fostering oxidative stress, mitochondrial damage, and apoptosis [24,25].

In line with these interpretations, therapeutic strategies targeting pyrimidine metabolism are currently under investigation. For instance, inhibitors of dihydroorotate dehydrogenase (DHODH), such as leflunomide and its derivatives, have demonstrated antiviral and immunomodulatory effects in preclinical and early clinical trials of COVID-19 [26,27]. By restricting de novo pyrimidine synthesis, they suppress viral replication and excessive immune responses. The enrichment of this pathway in severe cases underscores its biological importance and potential as a treatment target.

While previous studies have linked pyrimidine metabolism to viral replication and immune activation, our data specifically support pathway-level dysregulation in severe COVID-19 based on KEGG enrichment and measured nucleotide-related metabolites. Together, these findings indicate that pyrimidine metabolism alterations represent a late-stage metabolic signature of critical illness rather than a universal feature of SARS-CoV-2 infection.

### Severity-dependent trends of key metabolites

Beyond pathway-level alterations, several individual metabolites exhibited consistent, severity-dependent trends across COVID-19 stages. Early to moderate disease (COV1–COV3) was characterized by a marked downregulation of central carbon metabolism intermediates, including pyruvic acid, lactic acid, succinic acid, fumaric acid, malic acid, and glyceric acid, suggesting progressive impairment of glycolytic flux and tricarboxylic acid (TCA) cycle activity [28]. These alterations were already evident in COV1 and persisted through COV4, indicating early disruption of energy metabolism. Amino acid metabolism was also broadly affected, with consistent downregulation of branched-chain amino acids (valine, leucine), glucogenic amino acids (threonine, asparagine, glutamine), and intermediates of nitrogen metabolism (ornithine, taurine). In contrast, lipid-associated metabolites showed progressive upregulation with increasing disease severity. Lysophosphatidylcholines (LPCs), lysophosphatidylethanolamines (LPEs), sphingomyelins, and bile acid derivatives were elevated already in COV1–COV3 and further expanded in COV4. Severe disease was additionally characterized by increased acylcarnitines (e.g., palmitoylcarnitine, oleoylcarnitine, linoleyl carnitine), suggesting impaired mitochondrial β-oxidation and fatty acid transport [29]. Together, these findings indicate a stepwise metabolic rewiring from early disruption of central energy and amino acid metabolism toward lipid remodeling, mitochondrial dysfunction, and enhanced nucleotide turnover in critical COVID-19.

Previous metabolomics studies have proposed deoxycytidine, ureidopropionate, kynurenine, and acylcarnitines as predictors of COVID-19 disease outcomes [6]. However, we observed only partial overlap with these findings in the current cohort. Although acylcarnitine levels were significantly elevated in the most severe cases (COV4), consistent with mitochondrial dysfunction and aligning with the meta-analysis by Valdés et al. [30], metabolites related to deoxycytidine and kynurenine did not show a consistent, severity-dependent increase across COVID-19 stages, contrary to data from Almulla et al. [31]. Ureidopropionate was also not a significant differentiator. These discrepancies likely reflect differences in cohort size, clinical stratification, analytical platforms, and disease stage definitions. Our results suggest that although some metabolic signatures may be conserved, the predictive value of individual metabolites depends on context, specific cohort factors, and methodology.

### Consideration of sex-based differences

Sex-specific differences in COVID-19 severity and immune responses have been widely reported, with males generally experiencing worse clinical outcomes [32]. Although our cohort reflected the typical sex distribution of hospitalized patients, its limited size precluded statistically robust sex-stratified metabolomic analyses.

Nevertheless, several pathways identified in this study, including tryptophan–kynurenine metabolism, nucleotide biosynthesis, and mitochondrial energy metabolism, are known to be influenced by sex hormones and immune regulation. Estrogen signaling, for example, can modulate IDO1 activity and mitochondrial function, potentially affecting kynurenine production and energy metabolism during infection [33,34].

While definitive conclusions regarding sex-specific metabolic signatures cannot be drawn from the present dataset, our findings suggest that sex may modulate COVID-19–associated metabolic rewiring. Larger, sex-balanced and longitudinal studies will be required to clarify the clinical and biological significance of these effects.

## Conclusions

Our stratified metabolomic profiling of COVID-19 patient plasma revealed distinct, severity-associated metabolic signatures, highlighting progressive divergence from healthy controls. The most profound alterations were observed in the COV4 group, consistent with advanced systemic dysfunction.

A key finding was the consistent enrichment of phenylalanine metabolism in early and moderate cases (COV1–COV3), suggesting that aromatic amino acid dysregulation may serve as an early marker of immune activation and metabolic stress. In contrast, severe cases (COV4) showed marked enrichment of pyrimidine metabolism, reflecting increased nucleotide demand driven by viral replication and immune cell turnover.

These findings underscore a dynamic shift in metabolic pathways with disease progression, from amino acid disturbances in earlier stages to nucleotide biosynthetic reprogramming in severe disease. This trajectory not only enhances our understanding of COVID-19 pathogenesis but also suggests stage-specific biomarkers and therapeutic targets, supporting the clinical relevance of metabolomic profiling in infectious disease management. Especially, interindividual variability within metabolomic clusters, as observed in Fig 3C, may indicate early metabolic divergence predictive of distinct clinical trajectories. For instance, patients in COV1 who show more pronounced separation from controls may represent individuals at higher risk of progression. Although the present study was not longitudinal and therefore cannot confirm such associations, these findings may serve as a foundation for hypothesis generation in future predictive metabolomics research.

### Limitations and future directions

While our findings offer valuable insights, they are based on cross-sectional data and require validation in larger, longitudinal cohorts to capture the dynamics of metabolic changes over time. Future studies should include more patients to strengthen generalizability. Nonetheless, a strength of our study is that, even with only five patients per group, we were able to detect consistent and biologically meaningful differences across disease stages. Integration with transcriptomic and lipidomic data could further provide a more comprehensive systems-level understanding of SARS-CoV-2–induced perturbations.

In addition, the potential influence of pharmacological treatment and oxygen therapy on the observed metabolomic profiles cannot be completely excluded. All patients were managed under a uniform institutional protocol; however, subtle effects related to corticosteroid use, anticoagulant therapy, or differences in oxygen supplementation may have contributed to intergroup variability. Future studies involving larger cohorts and controlled treatment regimens will be required to disentangle these confounding factors. Furthermore, blood samples were collected at a single, early disease timepoint, within the first 2 hours of hospitalization, immediately after molecular confirmation of SARS-CoV-2 infection. Although this standardized approach minimized confounding due to treatment effects, it does not capture temporal metabolic shifts occurring throughout the disease course. Future longitudinal metabolomic studies, spanning multiple time points from early infection to recovery, will be essential to elucidate the dynamic trajectory of host metabolic reprogramming in COVID-19. Therefore, the observed metabolomic signatures should be interpreted with caution, as they may reflect a combination of disease-related and treatment-related effects.

Another limitation of this study is the unequal sex distribution within the study cohort (13 males and 7 females), which reflects the demographic pattern of hospitalized COVID-19 patients during the study period. Although sex-specific metabolic differences cannot be excluded, the limited cohort size did not allow for reliable subgroup analysis by sex. Future studies with larger, sex-balanced cohorts are needed to validate and extend our observations. Further, targeted validation of key metabolites using mass spectrometry and functional assays is warranted to confirm their role as prognostic or diagnostic biomarkers. Furthermore, because metabolite identification was based on putative annotations without validation with authentic standards, the results should be interpreted primarily at the pathway and systems levels.

## Supporting information

S1 File S1 Fig. The PCA score plot of the QC samples, the X axis indicates the number of QC samples, the Y axis indicates the range of RSD. **S2 Fig.** A volcano plot was generated to visualize the metabolite distribution for the CTR vs. COV1 comparison. The log2FC was plotted on the x-axis against the − log10(T-test p-value) on the y-axis. Metabolites were represented by circles for ESI− mode and triangles for ESI+ mode. Differential expression was indicated by color-coding: red and green were used for downregulated and upregulated metabolites, respectively, while gray was assigned to statistically non-significant features. The same metabolite may be detected in both ESI+ and ESI− modes and is therefore presented as separate analytical features. **S3 Fig.** The distribution of metabolites in the CTR vs. COV2 comparison was displayed using a volcano plot. While the x-axis reflected the log2FC, the y-axis represented the − log10(T-test p-value). Symbols were differentiated by ionization mode, with circles denoting ESI− and triangles denoting ESI + . Statistical significance was visualized through color-coding, where downregulated metabolites were marked in red, upregulated in green, and non-significant ones in gray. The same metabolite may be detected in both ESI+ and ESI− modes and is therefore presented as separate analytical features. **S4 Fig.** For the CTR vs. COV3 comparison, a volcano plot was constructed to show the metabolic profile. The relationship between log2FC (x-axis) and −log10(p-value) (y-axis) was examined. Ionization modes were depicted as circles (ESI−) and triangles (ESI+). Furthermore, metabolites were categorized by color: red was utilized for downregulation, green for upregulation, and gray for those that were found to be statistically non-significant. The same metabolite may be detected in both ESI+ and ESI− modes and is therefore presented as separate analytical features. **S5 Fig.** Metabolic variations between CTR and COV4 were illustrated via a volcano plot. The log2FC and the negative decadic logarithm of the p-value were assigned to the x and y axes, respectively. Circles and triangles were employed to distinguish between ESI− and ESI+ detections. A color scheme was applied to highlight significance: red for downregulation, green for upregulation, and gray for metabolites that were not considered statistically significant. The same metabolite may be detected in both ESI+ and ESI− modes and is therefore presented as separate analytical features. **S6 Fig.** Chemical classification of statistically significant metabolites from the CTR vs. COV1 comparison was summarized in a barplot. Chemical class annotations were plotted on the y-axis, while the count of involved metabolites was displayed on the x-axis. The coloration of the bars was determined by the FDR values, following the scale provided on the right side of the graph. **S7 Fig.** A barplot was utilized to represent the chemical hierarchy of significant metabolites identified in the CTR vs. COV2 group. The x-axis indicated the total number of metabolites per category, whereas the y-axis consisted of the chemical classification labels. Bar colors were assigned based on the FDR significance level, as shown in the legend. **S8 Fig.** Significant metabolites from the CTR vs. COV3 comparison were organized by chemical classification in this barplot. The distribution was mapped with classification annotations on the y-axis and metabolite counts on the x-axis. To reflect statistical confidence, bars were color-coded according to the FDR scale presented on the right. **S9 Fig.** The chemical diversity of statistically significant features in the CTR vs. COV4 comparison was analyzed through a barplot. The number of metabolites per class was recorded on the x-axis against their respective chemical annotations on the y-axis. Bars were filled with colors corresponding to their FDR values, according to the adjacent color gradient.
(ZIP)

S2 File Supplemental tables. (XLSX)
