# Correction: Severity-dependent metabolic rewiring in COVID-19 based on untargeted metabolomic profiling of patient plasma

**DOI:** 10.1371/journal.pone.0354449

**Published:** 2026-07-22

**Authors:** Marta Majewska, Mateusz A. Maździarz, Ewa Lepiarczyk, Aleksandra Lipka, Marta Wiszpolska, Beata Moczulska, Elżbieta Łopieńska-Biernat, Piotr Iwanowicz, Piotr Kocbach, Hilde Galtung, Leszek Gromadziński

Fig 4 was uploaded incorrectly. Please see the correct Fig 4 here.

**Fig 4:**
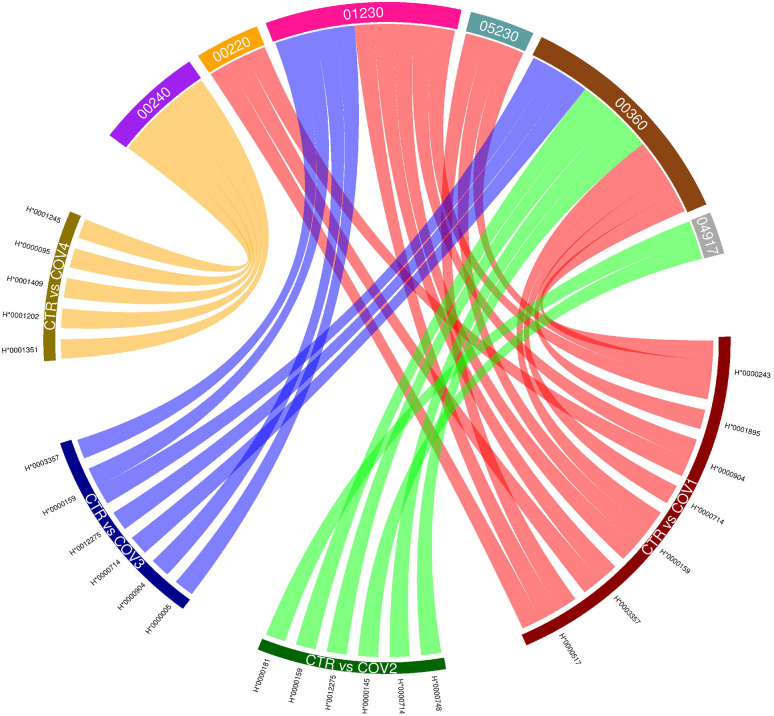
The chord diagram illustrates the statistically significant KEGG pathways identified in several comparisons.
