# Supplementary figures and images for: Severity-dependent metabolic rewiring in COVID-19 based on untargeted metabolomic profiling of patient plasma
Source: PLoS One. 2026 Jun 25;21(6):e0352437. doi: 10.1371/journal.pone.0352437 (PMC13298738; doi:10.1371/journal.pone.0352437)

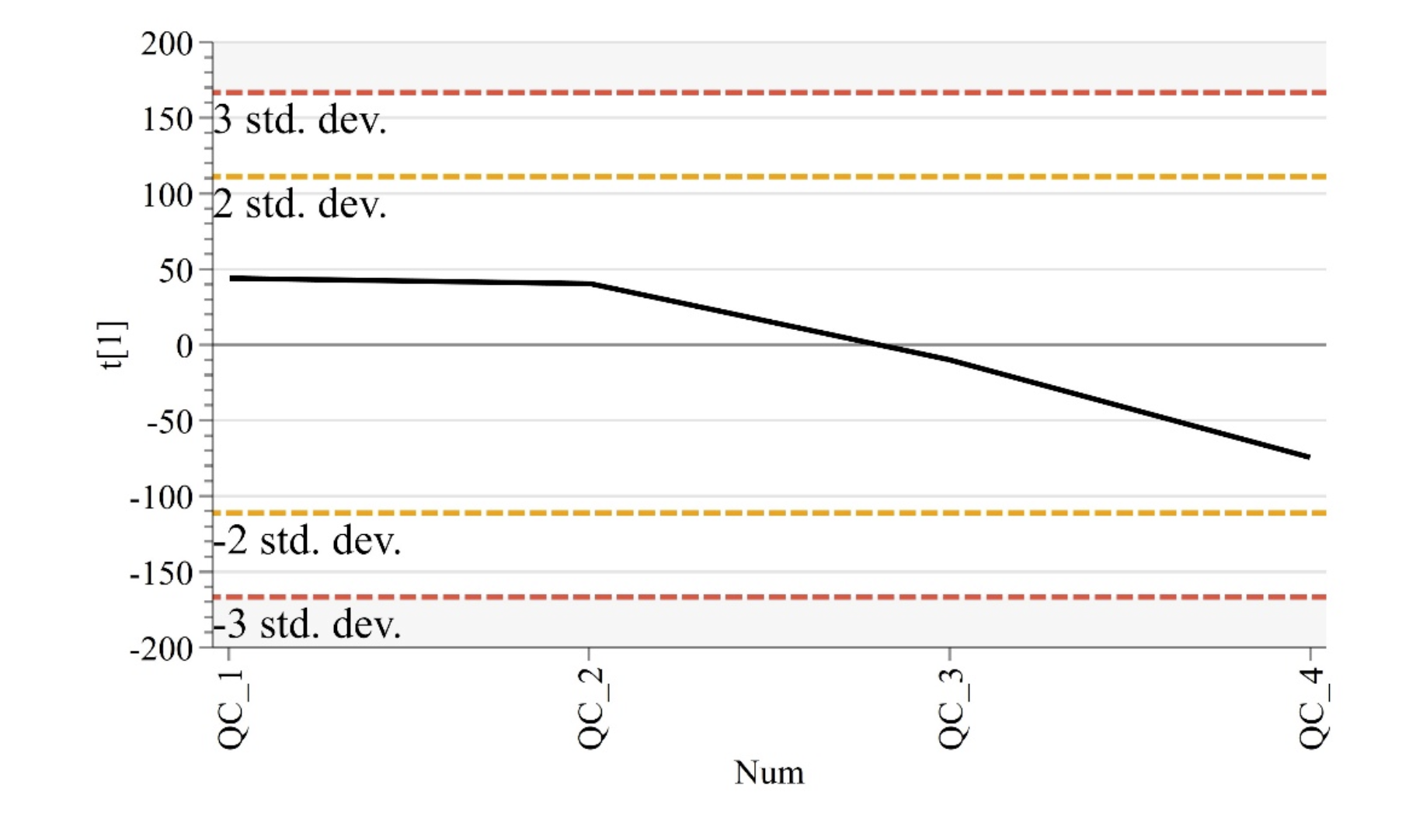

Supplement: S1 File — S2 Fig. A volcano plot was generated to visualize the metabolite distribution for the CTR vs. COV1 comparison. The log2FC was plotted on the x-axis against the − log10(T-test p-value) on the y-axis. Metabolites were represented by circles for ESI− mode and triangles for ESI+ mode. Differential expression was indicated by color-coding: red and green were used for downregulated and upregulated metabolites, respectively, while gray was assigned to statistically non-significant features. The same metabolite may be detected in both ESI+ and ESI− modes and is therefore presented as separate analytical features. S3 Fig. The distribution of metabolites in the CTR vs. COV2 comparison was displayed using a volcano plot. While the x-axis reflected the log2FC, the y-axis represented the − log10(T-test p-value). Symbols were differentiated by ionization mode, with circles denoting ESI− and triangles denoting ESI + . Statistical significance was visualized through color-coding, where downregulated metabolites were marked in red, upregulated in green, and non-significant ones in gray. The same metabolite may be detected in both ESI+ and ESI− modes and is therefore presented as separate analytical features. S4 Fig. For the CTR vs. COV3 comparison, a volcano plot was constructed to show the metabolic profile. The relationship between log2FC (x-axis) and −log10(p-value) (y-axis) was examined. Ionization modes were depicted as circles (ESI−) and triangles (ESI+). Furthermore, metabolites were categorized by color: red was utilized for downregulation, green for upregulation, and gray for those that were found to be statistically non-significant. The same metabolite may be detected in both ESI+ and ESI− modes and is therefore presented as separate analytical features. S5 Fig. Metabolic variations between CTR and COV4 were illustrated via a volcano plot. The log2FC and the negative decadic logarithm of the p-value were assigned to the x and y axes, respectively. Circles and triangles wer [file pone.0352437.s001.zip › S1_fig.png]

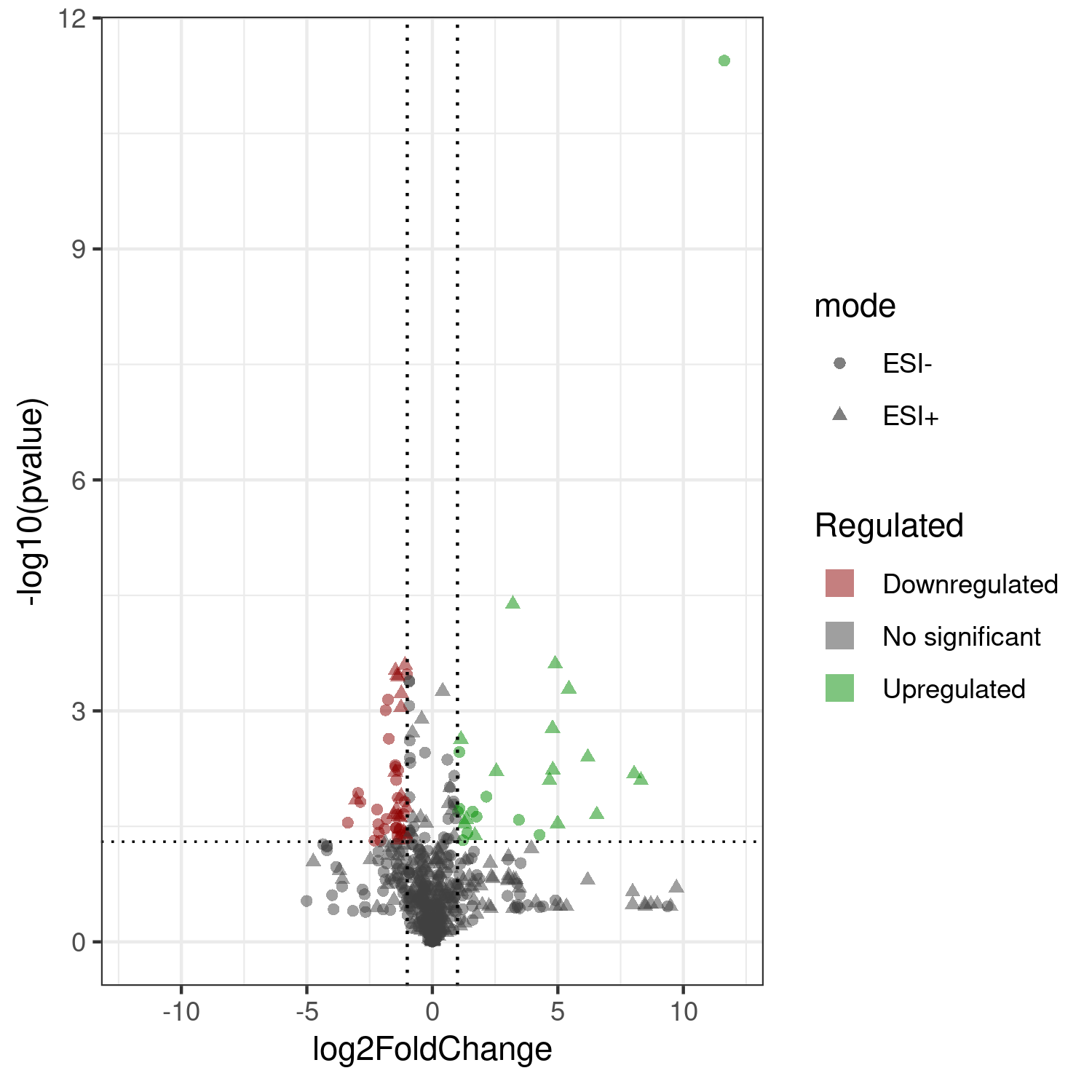

Supplement: S1 File — S2 Fig. A volcano plot was generated to visualize the metabolite distribution for the CTR vs. COV1 comparison. The log2FC was plotted on the x-axis against the − log10(T-test p-value) on the y-axis. Metabolites were represented by circles for ESI− mode and triangles for ESI+ mode. Differential expression was indicated by color-coding: red and green were used for downregulated and upregulated metabolites, respectively, while gray was assigned to statistically non-significant features. The same metabolite may be detected in both ESI+ and ESI− modes and is therefore presented as separate analytical features. S3 Fig. The distribution of metabolites in the CTR vs. COV2 comparison was displayed using a volcano plot. While the x-axis reflected the log2FC, the y-axis represented the − log10(T-test p-value). Symbols were differentiated by ionization mode, with circles denoting ESI− and triangles denoting ESI + . Statistical significance was visualized through color-coding, where downregulated metabolites were marked in red, upregulated in green, and non-significant ones in gray. The same metabolite may be detected in both ESI+ and ESI− modes and is therefore presented as separate analytical features. S4 Fig. For the CTR vs. COV3 comparison, a volcano plot was constructed to show the metabolic profile. The relationship between log2FC (x-axis) and −log10(p-value) (y-axis) was examined. Ionization modes were depicted as circles (ESI−) and triangles (ESI+). Furthermore, metabolites were categorized by color: red was utilized for downregulation, green for upregulation, and gray for those that were found to be statistically non-significant. The same metabolite may be detected in both ESI+ and ESI− modes and is therefore presented as separate analytical features. S5 Fig. Metabolic variations between CTR and COV4 were illustrated via a volcano plot. The log2FC and the negative decadic logarithm of the p-value were assigned to the x and y axes, respectively. Circles and triangles wer [file pone.0352437.s001.zip › S2_fig.png]

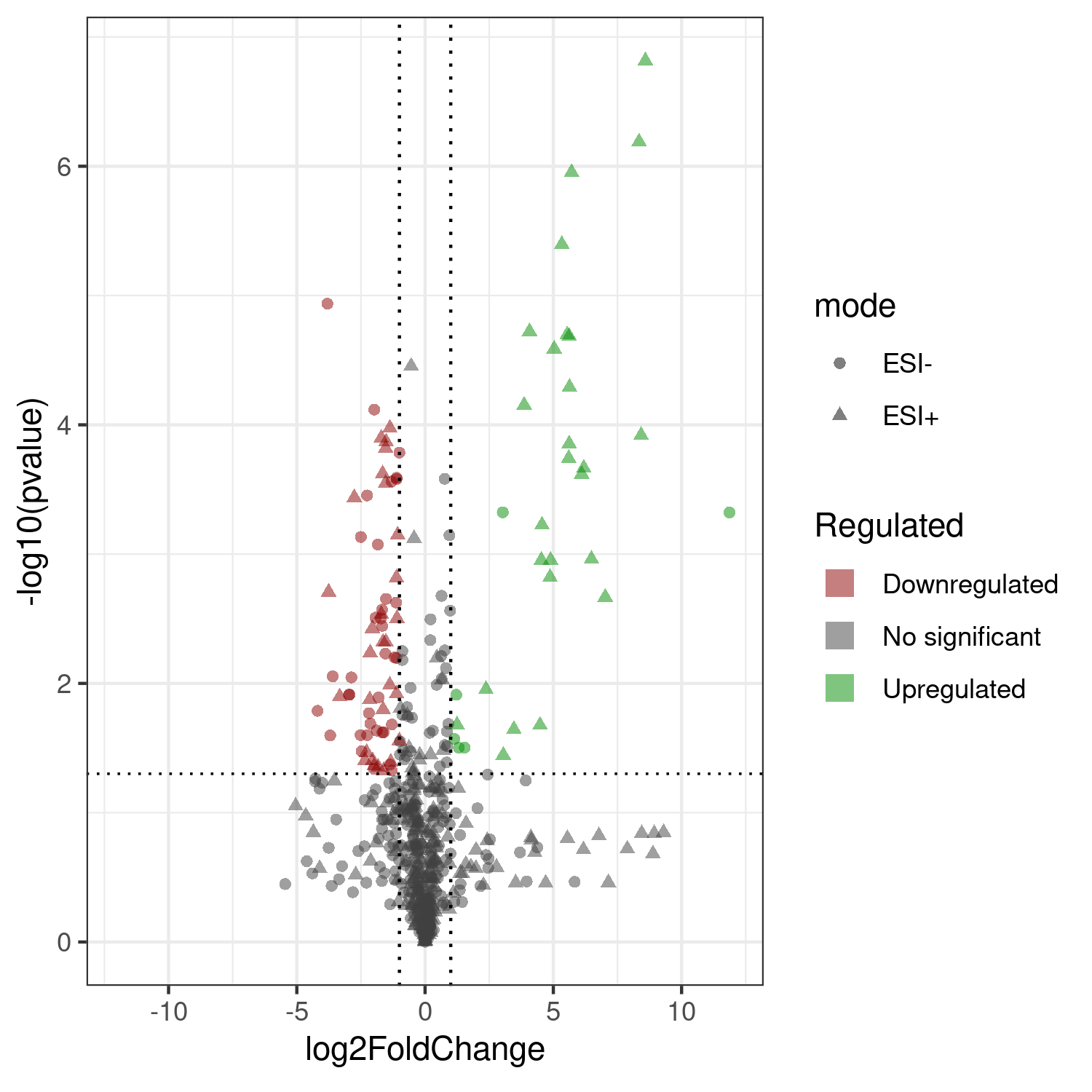

Supplement: S1 File — S2 Fig. A volcano plot was generated to visualize the metabolite distribution for the CTR vs. COV1 comparison. The log2FC was plotted on the x-axis against the − log10(T-test p-value) on the y-axis. Metabolites were represented by circles for ESI− mode and triangles for ESI+ mode. Differential expression was indicated by color-coding: red and green were used for downregulated and upregulated metabolites, respectively, while gray was assigned to statistically non-significant features. The same metabolite may be detected in both ESI+ and ESI− modes and is therefore presented as separate analytical features. S3 Fig. The distribution of metabolites in the CTR vs. COV2 comparison was displayed using a volcano plot. While the x-axis reflected the log2FC, the y-axis represented the − log10(T-test p-value). Symbols were differentiated by ionization mode, with circles denoting ESI− and triangles denoting ESI + . Statistical significance was visualized through color-coding, where downregulated metabolites were marked in red, upregulated in green, and non-significant ones in gray. The same metabolite may be detected in both ESI+ and ESI− modes and is therefore presented as separate analytical features. S4 Fig. For the CTR vs. COV3 comparison, a volcano plot was constructed to show the metabolic profile. The relationship between log2FC (x-axis) and −log10(p-value) (y-axis) was examined. Ionization modes were depicted as circles (ESI−) and triangles (ESI+). Furthermore, metabolites were categorized by color: red was utilized for downregulation, green for upregulation, and gray for those that were found to be statistically non-significant. The same metabolite may be detected in both ESI+ and ESI− modes and is therefore presented as separate analytical features. S5 Fig. Metabolic variations between CTR and COV4 were illustrated via a volcano plot. The log2FC and the negative decadic logarithm of the p-value were assigned to the x and y axes, respectively. Circles and triangles wer [file pone.0352437.s001.zip › S3_fig.png]

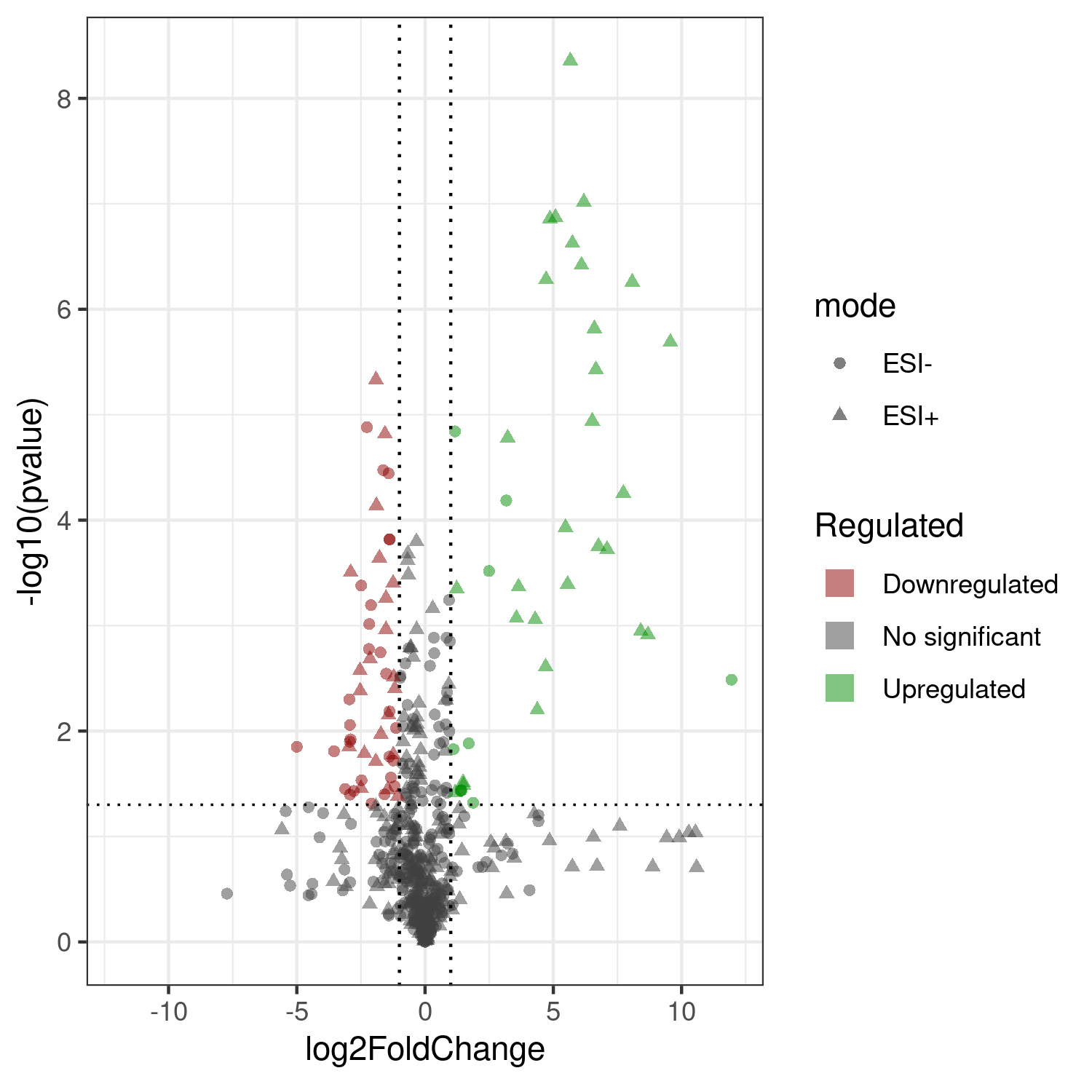

Supplement: S1 File — S2 Fig. A volcano plot was generated to visualize the metabolite distribution for the CTR vs. COV1 comparison. The log2FC was plotted on the x-axis against the − log10(T-test p-value) on the y-axis. Metabolites were represented by circles for ESI− mode and triangles for ESI+ mode. Differential expression was indicated by color-coding: red and green were used for downregulated and upregulated metabolites, respectively, while gray was assigned to statistically non-significant features. The same metabolite may be detected in both ESI+ and ESI− modes and is therefore presented as separate analytical features. S3 Fig. The distribution of metabolites in the CTR vs. COV2 comparison was displayed using a volcano plot. While the x-axis reflected the log2FC, the y-axis represented the − log10(T-test p-value). Symbols were differentiated by ionization mode, with circles denoting ESI− and triangles denoting ESI + . Statistical significance was visualized through color-coding, where downregulated metabolites were marked in red, upregulated in green, and non-significant ones in gray. The same metabolite may be detected in both ESI+ and ESI− modes and is therefore presented as separate analytical features. S4 Fig. For the CTR vs. COV3 comparison, a volcano plot was constructed to show the metabolic profile. The relationship between log2FC (x-axis) and −log10(p-value) (y-axis) was examined. Ionization modes were depicted as circles (ESI−) and triangles (ESI+). Furthermore, metabolites were categorized by color: red was utilized for downregulation, green for upregulation, and gray for those that were found to be statistically non-significant. The same metabolite may be detected in both ESI+ and ESI− modes and is therefore presented as separate analytical features. S5 Fig. Metabolic variations between CTR and COV4 were illustrated via a volcano plot. The log2FC and the negative decadic logarithm of the p-value were assigned to the x and y axes, respectively. Circles and triangles wer [file pone.0352437.s001.zip › S4_fig.png]

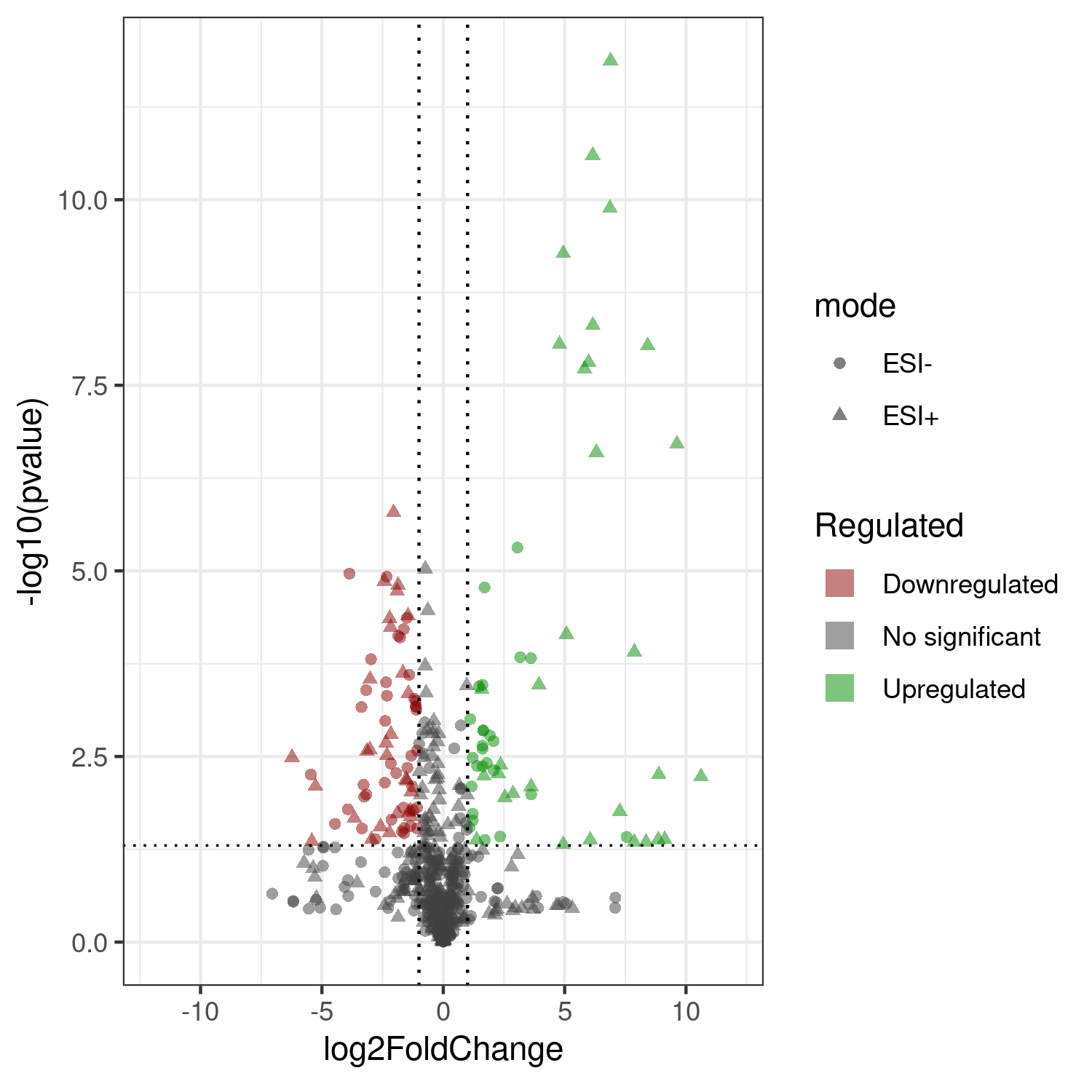

Supplement: S1 File — S2 Fig. A volcano plot was generated to visualize the metabolite distribution for the CTR vs. COV1 comparison. The log2FC was plotted on the x-axis against the − log10(T-test p-value) on the y-axis. Metabolites were represented by circles for ESI− mode and triangles for ESI+ mode. Differential expression was indicated by color-coding: red and green were used for downregulated and upregulated metabolites, respectively, while gray was assigned to statistically non-significant features. The same metabolite may be detected in both ESI+ and ESI− modes and is therefore presented as separate analytical features. S3 Fig. The distribution of metabolites in the CTR vs. COV2 comparison was displayed using a volcano plot. While the x-axis reflected the log2FC, the y-axis represented the − log10(T-test p-value). Symbols were differentiated by ionization mode, with circles denoting ESI− and triangles denoting ESI + . Statistical significance was visualized through color-coding, where downregulated metabolites were marked in red, upregulated in green, and non-significant ones in gray. The same metabolite may be detected in both ESI+ and ESI− modes and is therefore presented as separate analytical features. S4 Fig. For the CTR vs. COV3 comparison, a volcano plot was constructed to show the metabolic profile. The relationship between log2FC (x-axis) and −log10(p-value) (y-axis) was examined. Ionization modes were depicted as circles (ESI−) and triangles (ESI+). Furthermore, metabolites were categorized by color: red was utilized for downregulation, green for upregulation, and gray for those that were found to be statistically non-significant. The same metabolite may be detected in both ESI+ and ESI− modes and is therefore presented as separate analytical features. S5 Fig. Metabolic variations between CTR and COV4 were illustrated via a volcano plot. The log2FC and the negative decadic logarithm of the p-value were assigned to the x and y axes, respectively. Circles and triangles wer [file pone.0352437.s001.zip › S5_fig.png]

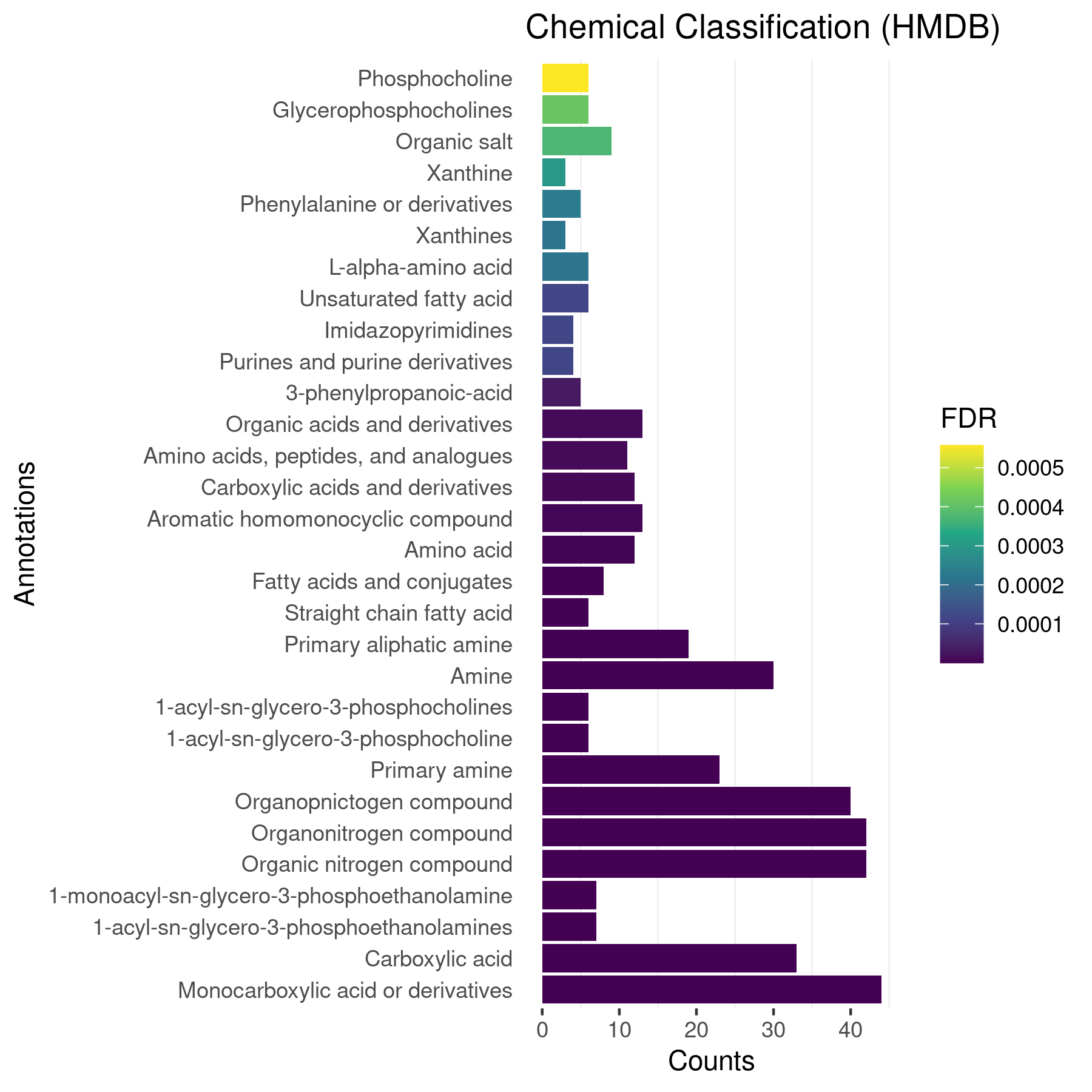

Supplement: S1 File — S2 Fig. A volcano plot was generated to visualize the metabolite distribution for the CTR vs. COV1 comparison. The log2FC was plotted on the x-axis against the − log10(T-test p-value) on the y-axis. Metabolites were represented by circles for ESI− mode and triangles for ESI+ mode. Differential expression was indicated by color-coding: red and green were used for downregulated and upregulated metabolites, respectively, while gray was assigned to statistically non-significant features. The same metabolite may be detected in both ESI+ and ESI− modes and is therefore presented as separate analytical features. S3 Fig. The distribution of metabolites in the CTR vs. COV2 comparison was displayed using a volcano plot. While the x-axis reflected the log2FC, the y-axis represented the − log10(T-test p-value). Symbols were differentiated by ionization mode, with circles denoting ESI− and triangles denoting ESI + . Statistical significance was visualized through color-coding, where downregulated metabolites were marked in red, upregulated in green, and non-significant ones in gray. The same metabolite may be detected in both ESI+ and ESI− modes and is therefore presented as separate analytical features. S4 Fig. For the CTR vs. COV3 comparison, a volcano plot was constructed to show the metabolic profile. The relationship between log2FC (x-axis) and −log10(p-value) (y-axis) was examined. Ionization modes were depicted as circles (ESI−) and triangles (ESI+). Furthermore, metabolites were categorized by color: red was utilized for downregulation, green for upregulation, and gray for those that were found to be statistically non-significant. The same metabolite may be detected in both ESI+ and ESI− modes and is therefore presented as separate analytical features. S5 Fig. Metabolic variations between CTR and COV4 were illustrated via a volcano plot. The log2FC and the negative decadic logarithm of the p-value were assigned to the x and y axes, respectively. Circles and triangles wer [file pone.0352437.s001.zip › S6_fig.png]

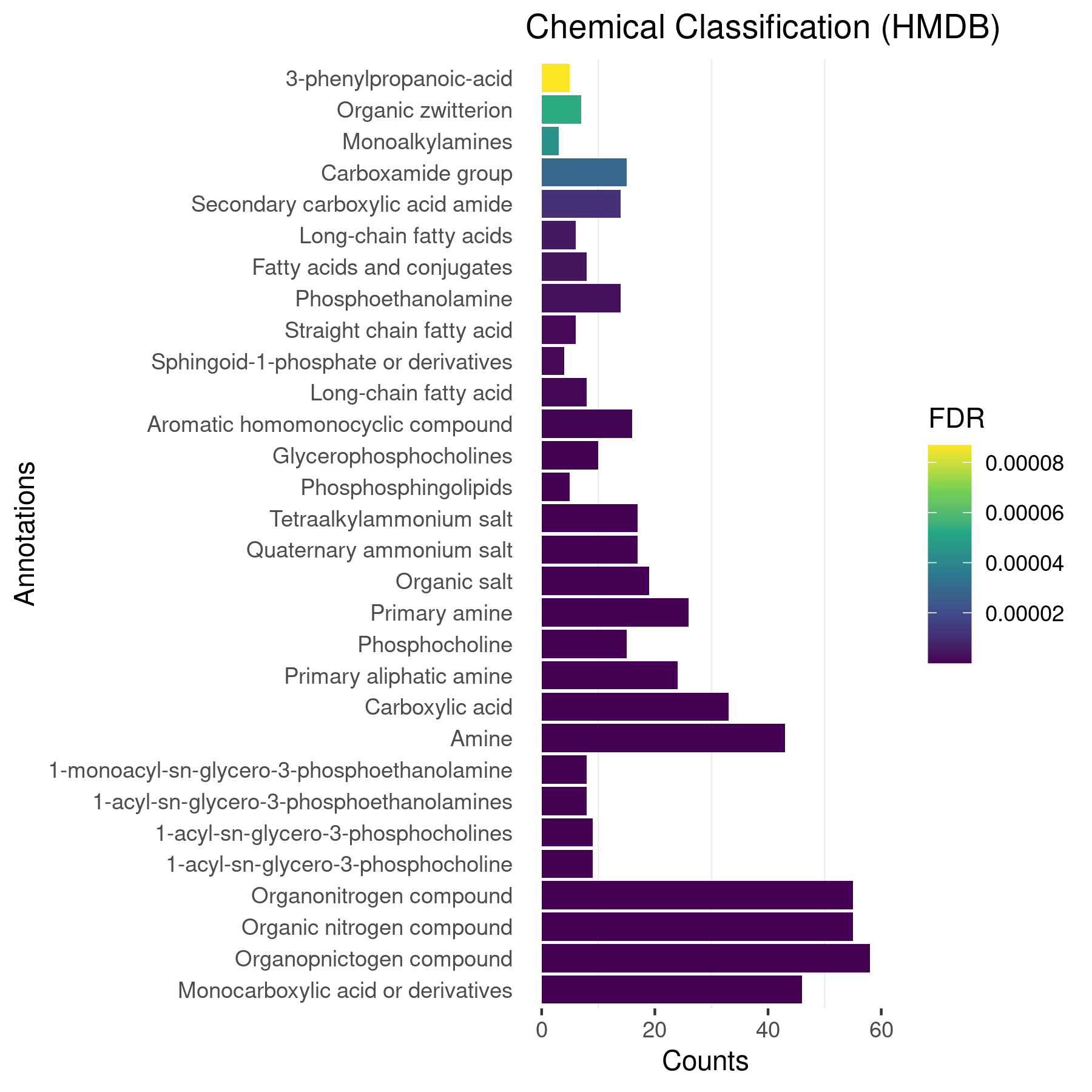

Supplement: S1 File — S2 Fig. A volcano plot was generated to visualize the metabolite distribution for the CTR vs. COV1 comparison. The log2FC was plotted on the x-axis against the − log10(T-test p-value) on the y-axis. Metabolites were represented by circles for ESI− mode and triangles for ESI+ mode. Differential expression was indicated by color-coding: red and green were used for downregulated and upregulated metabolites, respectively, while gray was assigned to statistically non-significant features. The same metabolite may be detected in both ESI+ and ESI− modes and is therefore presented as separate analytical features. S3 Fig. The distribution of metabolites in the CTR vs. COV2 comparison was displayed using a volcano plot. While the x-axis reflected the log2FC, the y-axis represented the − log10(T-test p-value). Symbols were differentiated by ionization mode, with circles denoting ESI− and triangles denoting ESI + . Statistical significance was visualized through color-coding, where downregulated metabolites were marked in red, upregulated in green, and non-significant ones in gray. The same metabolite may be detected in both ESI+ and ESI− modes and is therefore presented as separate analytical features. S4 Fig. For the CTR vs. COV3 comparison, a volcano plot was constructed to show the metabolic profile. The relationship between log2FC (x-axis) and −log10(p-value) (y-axis) was examined. Ionization modes were depicted as circles (ESI−) and triangles (ESI+). Furthermore, metabolites were categorized by color: red was utilized for downregulation, green for upregulation, and gray for those that were found to be statistically non-significant. The same metabolite may be detected in both ESI+ and ESI− modes and is therefore presented as separate analytical features. S5 Fig. Metabolic variations between CTR and COV4 were illustrated via a volcano plot. The log2FC and the negative decadic logarithm of the p-value were assigned to the x and y axes, respectively. Circles and triangles wer [file pone.0352437.s001.zip › S7_fig.png]

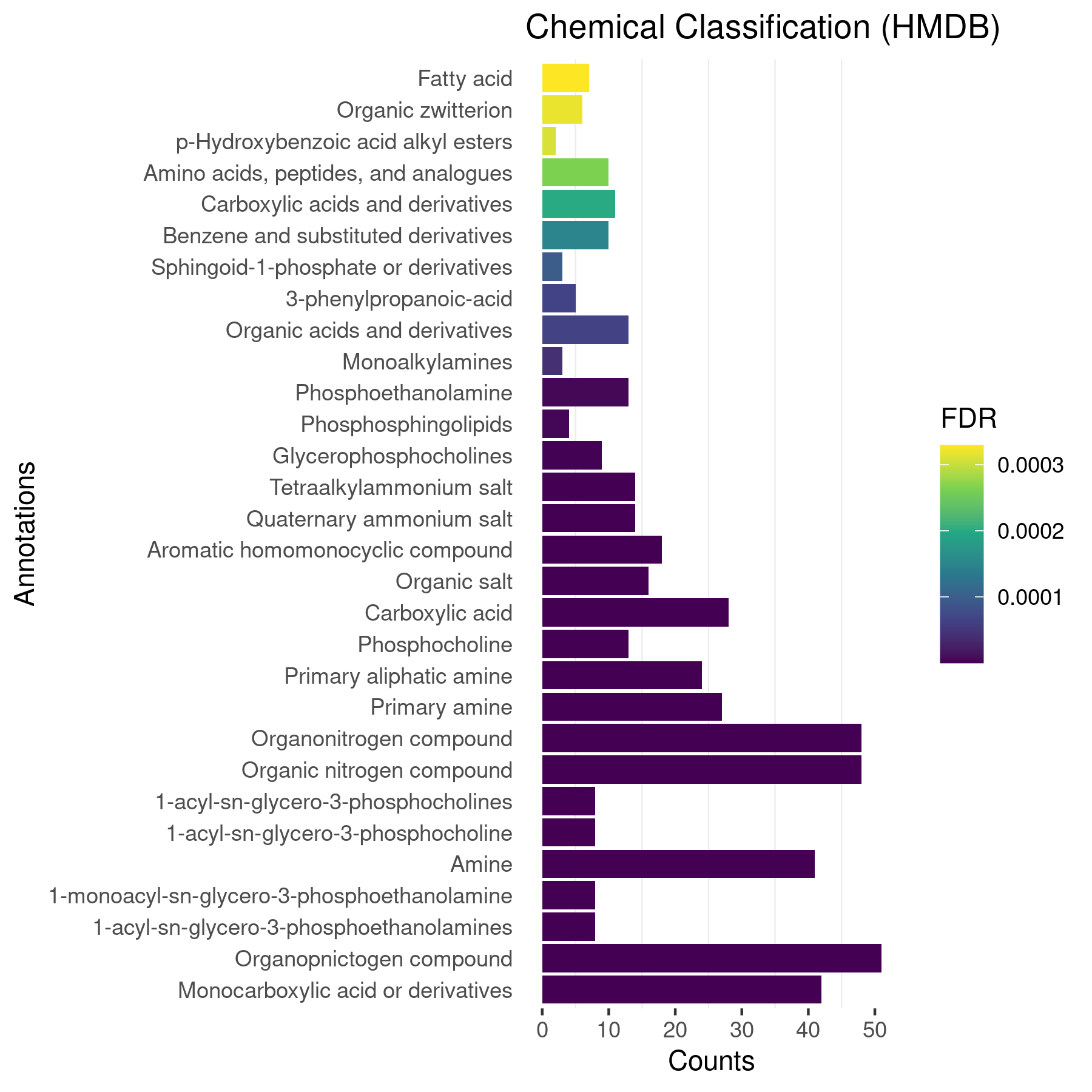

Supplement: S1 File — S2 Fig. A volcano plot was generated to visualize the metabolite distribution for the CTR vs. COV1 comparison. The log2FC was plotted on the x-axis against the − log10(T-test p-value) on the y-axis. Metabolites were represented by circles for ESI− mode and triangles for ESI+ mode. Differential expression was indicated by color-coding: red and green were used for downregulated and upregulated metabolites, respectively, while gray was assigned to statistically non-significant features. The same metabolite may be detected in both ESI+ and ESI− modes and is therefore presented as separate analytical features. S3 Fig. The distribution of metabolites in the CTR vs. COV2 comparison was displayed using a volcano plot. While the x-axis reflected the log2FC, the y-axis represented the − log10(T-test p-value). Symbols were differentiated by ionization mode, with circles denoting ESI− and triangles denoting ESI + . Statistical significance was visualized through color-coding, where downregulated metabolites were marked in red, upregulated in green, and non-significant ones in gray. The same metabolite may be detected in both ESI+ and ESI− modes and is therefore presented as separate analytical features. S4 Fig. For the CTR vs. COV3 comparison, a volcano plot was constructed to show the metabolic profile. The relationship between log2FC (x-axis) and −log10(p-value) (y-axis) was examined. Ionization modes were depicted as circles (ESI−) and triangles (ESI+). Furthermore, metabolites were categorized by color: red was utilized for downregulation, green for upregulation, and gray for those that were found to be statistically non-significant. The same metabolite may be detected in both ESI+ and ESI− modes and is therefore presented as separate analytical features. S5 Fig. Metabolic variations between CTR and COV4 were illustrated via a volcano plot. The log2FC and the negative decadic logarithm of the p-value were assigned to the x and y axes, respectively. Circles and triangles wer [file pone.0352437.s001.zip › S8_fig.png]

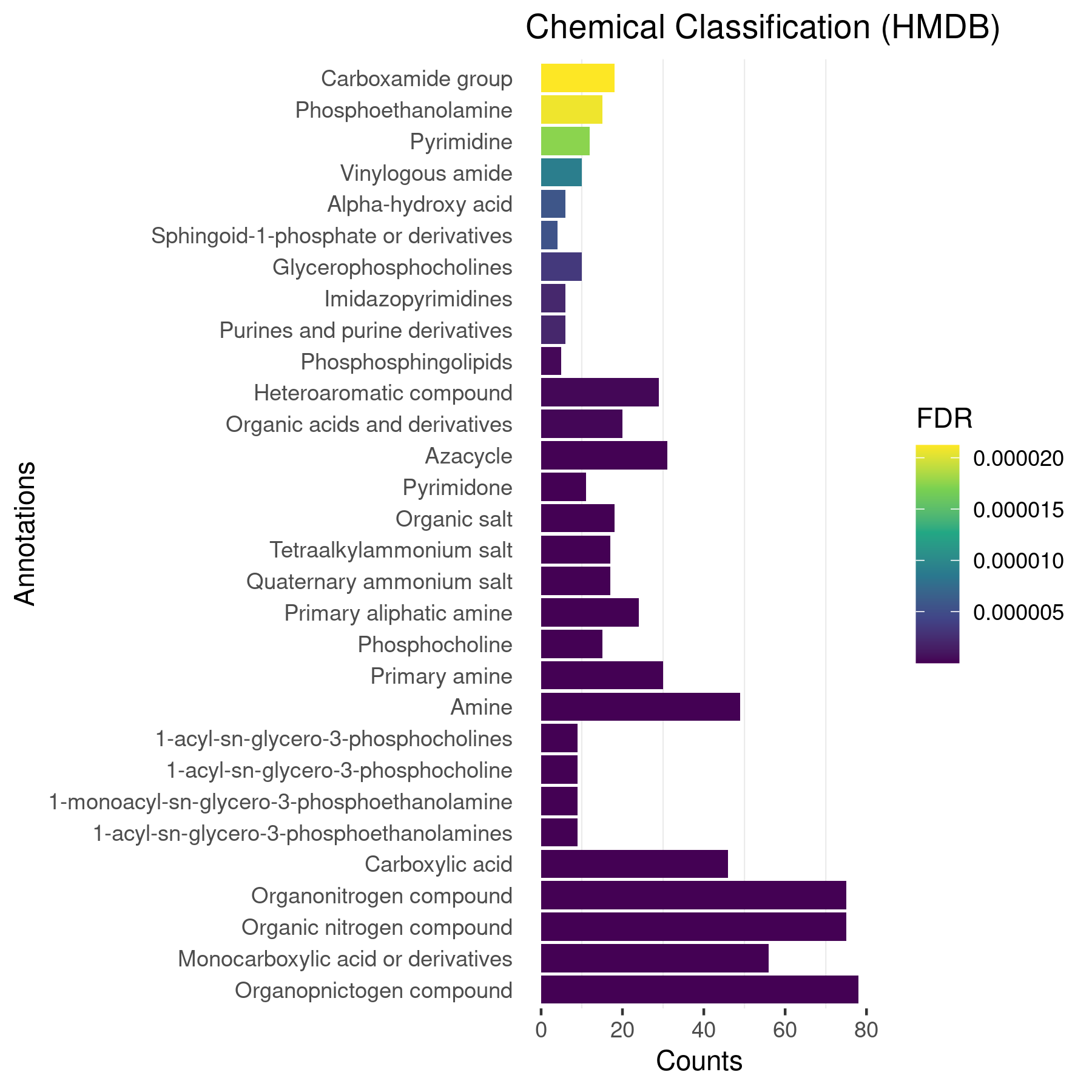

Supplement: S1 File — S2 Fig. A volcano plot was generated to visualize the metabolite distribution for the CTR vs. COV1 comparison. The log2FC was plotted on the x-axis against the − log10(T-test p-value) on the y-axis. Metabolites were represented by circles for ESI− mode and triangles for ESI+ mode. Differential expression was indicated by color-coding: red and green were used for downregulated and upregulated metabolites, respectively, while gray was assigned to statistically non-significant features. The same metabolite may be detected in both ESI+ and ESI− modes and is therefore presented as separate analytical features. S3 Fig. The distribution of metabolites in the CTR vs. COV2 comparison was displayed using a volcano plot. While the x-axis reflected the log2FC, the y-axis represented the − log10(T-test p-value). Symbols were differentiated by ionization mode, with circles denoting ESI− and triangles denoting ESI + . Statistical significance was visualized through color-coding, where downregulated metabolites were marked in red, upregulated in green, and non-significant ones in gray. The same metabolite may be detected in both ESI+ and ESI− modes and is therefore presented as separate analytical features. S4 Fig. For the CTR vs. COV3 comparison, a volcano plot was constructed to show the metabolic profile. The relationship between log2FC (x-axis) and −log10(p-value) (y-axis) was examined. Ionization modes were depicted as circles (ESI−) and triangles (ESI+). Furthermore, metabolites were categorized by color: red was utilized for downregulation, green for upregulation, and gray for those that were found to be statistically non-significant. The same metabolite may be detected in both ESI+ and ESI− modes and is therefore presented as separate analytical features. S5 Fig. Metabolic variations between CTR and COV4 were illustrated via a volcano plot. The log2FC and the negative decadic logarithm of the p-value were assigned to the x and y axes, respectively. Circles and triangles wer [file pone.0352437.s001.zip › S9_fig.png]
